# Developments in Ion Exchange Chromatography–Mass Spectrometry for the Characterization of Intact Proteins and Proteoforms

**DOI:** 10.1002/jssc.70268

**Published:** 2025-09-21

**Authors:** Ziran Zhai, Thomas Holmark, Lars Jasperse, Andrea F. G. Gargano

**Affiliations:** ^1^ Analytical Chemistry Group Van't Hoff Institute for Molecular Sciences (HIMS) University of Amsterdam Amsterdam the Netherlands; ^2^ Centre for Analytical Sciences Amsterdam Van't Hoff Institute for Molecular Sciences (HIMS) University of Amsterdam Amsterdam the Netherlands

**Keywords:** charge variants, intact protein analysis, ion exchange chromatography, mass spectrometry, pH gradient

## Abstract

Ion exchange chromatography (IEC) hyphenated to mass spectrometry (MS) is a powerful non‐denaturing technique used for analyzing protein charge heterogeneity (including posttranslational modifications such as deamidation, phosphorylation, and sialylation), as well as for characterizing complex protein mixtures. This review provides an overview of current strategies for implementing IEC–MS, focusing on pH gradient‐based methods: chromatofocusing, linear pH gradient elution, and salt‐mediated pH gradient elution. First, the fundamental principles, elution modes, and separation mechanisms of IEC are introduced. The review then discusses the limitations of traditional salt gradient elution in IEC, which often relies on nonvolatile additives, and highlights the critical role of pH gradient‐based IEC in enabling direct and efficient hyphenation with MS. Key factors influencing protein elution in IEC are summarized to aid method optimization and enhance the understanding of the separation process. Subsequently, recent advancements in IEC–MS are described, covering three key aspects: (i) the selection of appropriate volatile buffer systems and strategies for achieving controlled linear pH gradients, (ii) developments and selection criteria for IEC columns, and (iii) approaches to improve sensitivity and ionization efficiency in IEC–MS. Finally, the review compiles details of recently developed (2010–2025) pH gradient‐based IEC–MS methods and summarizes their applications in identifying diverse therapeutic protein variants arising from PTMs, antibody‐drug conjugates (ADCs), and other complex biological samples.

**Abbreviations**
^1^D: first dimension
^2^D: second dimension
2DLC: two‐dimensional liquid chromatography
AA: acetic acid
AAV: adeno‐associated virus
Aces: *N*‐(2‐acetamido)‐2‐aminoethanesulfonic acid
ACN: acetonitrile
ADC: antibody‐drug conjugate
AEC or AEX: anion exchange chromatography
AF4: asymmetric flow field‐flow fractionation
AGP: alpha‐1‐acid glycoprotein
AmAc: ammonium acetate
AmCa: ammonium carbonate
AmBc: ammonium bicarbonate
AmFo: Ammonium formate
AmHy: ammonium hydroxide
BMEP: bis[2‐(methacryloyloxy)ethyl] phosphate
BSA: bovine serum albumin
BsAbs: bispecific antibodies
CA: carbonic anhydrase
CEC or CEX: cation exchange chromatography
CFA: co‐formulate antibodies
CPA: colloidal particle adsorption model
CQA: critical quality attribute
CZE: capillary zone electrophoresis
DEN: dopant‐enhanced nitrogen
DFEA: 2,2‐difluoroethylamine
DIX: Donnan ion exchange model
DMSO: dimethyl sulfoxide
DVB: divinylbenzene
EPO: erythropoietin
ESI–Q–ToF–MS: electrospray ionization–quadrupole–time of flight–mass spectrometry
EtOH: ethanol
EVB: ethylvinylbenzene
FA: formic acid
FLD: Fluorescence detector
GalNAc: *N*‐acetylgalactosamine
HC: heavy chain
HCl: hydrochloric acid
HDL: high‐density lipoprotein
HEPES: 4‐2‐hydroxyethyl‐1‐piperazineethanesulfonic acid
HIC: hydrophobic interaction chromatography
HILIC: Hydrophilic interaction liquid chromatography
IEC: ion exchange chromatography
IgE: immunoglobulin subclass‐E
IgG: immunoglobulin subclass‐G
isoAsp: isoaspartate
IPA: isopropyl alcohol
LC: liquid chromatography or light chain
LGE: linear gradient elution model
LSS: linear solvent strength
mAb: monoclonal antibody
MES: 2‐(*N*‐morpholino) methanesulfonic acid
MeOH: methanol
MS: mass spectrometry
M3: microfabricated monolithic multinozzle
MW: molecular weight
NaCl: sodium chloride
Neu5Ac: *N*‐acetylneuraminic acid
Neu5Gc: *N*‐glycolylneuraminic
nMS: native mass spectrometry
SPs: stationary phases
OPN: osetopontin
PAHEMA: phosphoric acid 2‐hydroxyethyl methacrylate
pE: pyroglutamate
PEGA: polyethylene glycol acrylate
PEGDA: polyethylene glycol diacrylate
pI: isoelectric point
PS: polystyrene
PTM: posttranslational modification
RPLC: reverse‐phase liquid chromatography
sdAbs: single domain antibodies
SAX: strong anion exchange chromatography
SCX: strong cation exchange chromatography
SEC: size‐exclusion chromatography
SMA: steric mass action model
TEAC: tetraethylammonium chloride
TFEA: 2,2,2‐trifluoroethylamine
Tris: tris‐(hydroxymethyl)‐aminomethane
TMAC: tetramethylammonium chloride
UGT: uridine diphosphoglucuronosyl transferase
UV: ultraviolet
VP: viral particle
WAX: weak anion exchange chromatography
WCX: weak cation exchange chromatography.

## 1 Introduction

Proteins are fundamental biomolecules with a wide variety of structural and functional variations, driving virtually all biological processes, from acting as enzymes and structural components to facilitating signaling and transport [1, 2]. Advances in biotechnology have enabled the recombinant production of proteins, allowing their broad application across various fields, most notably in the development of biopharmaceuticals [3, 4].

Proteins exist in various forms, known as proteoforms, which arise from genetic variations, alternative splicing, and posttranslational modifications (PTMs) [5, 6, 7, 8]. Characterizing proteoforms at the intact protein level and using top‐down analysis (combining intact protein mass results with protein analysis by MS/MS fragmentation) is crucial for understanding their structural diversity, allowing the characterization of proteoform distributions. However, intact protein analysis presents substantial challenges in both the characterization of purified protein products—such as biopharmaceuticals (e.g., monoclonal antibodies [mAbs])—and in the analysis of complex protein mixtures (e.g., proteins contained in biofluids or cell lysates), due to the large molecular size (typically tens to hundreds of kDa) and considerable heterogeneity (e.g., sequence variants, PTMs, and formation of protein complexes) [9, 10]. Although direct‐injection mass spectrometry (MS) can be performed on (highly) purified samples, coupling liquid chromatography (LC) to MS (LC–MS) can resolve proteoforms in the liquid phase, allowing the study of samples with higher proteoform complexity and characterizing less abundant proteoforms [11, 12, 13].

A key challenge in LC–MS analysis of intact proteins lies in achieving high‐resolution separations under conditions compatible with MS. Moreover, there is growing interest in performing protein analysis by LC–MS under non‐denaturing conditions, as this approach can (i) facilitate MS analysis by maintaining the native protein state, which typically results in a limited number of charge states—even for large proteins—often shifted to higher *m*/*z* values; and (ii) preserve non‐covalent interactions, which are crucial for maintaining protein structure and function [1, 14]. The need for native MS‐hyphenated separations has led to the development of methods such as size‐exclusion chromatography (SEC) [15, 16] and asymmetric flow field‐flow fractionation (AF4) [17] (both separating proteins by size or hydrodynamic radius), hydrophobic‐interaction chromatography (HIC) [18] (by hydrophobicity), and native capillary zone electrophoresis (CZE) [19] (by charge‐to‐size ratio).

**FIGURE 1:**
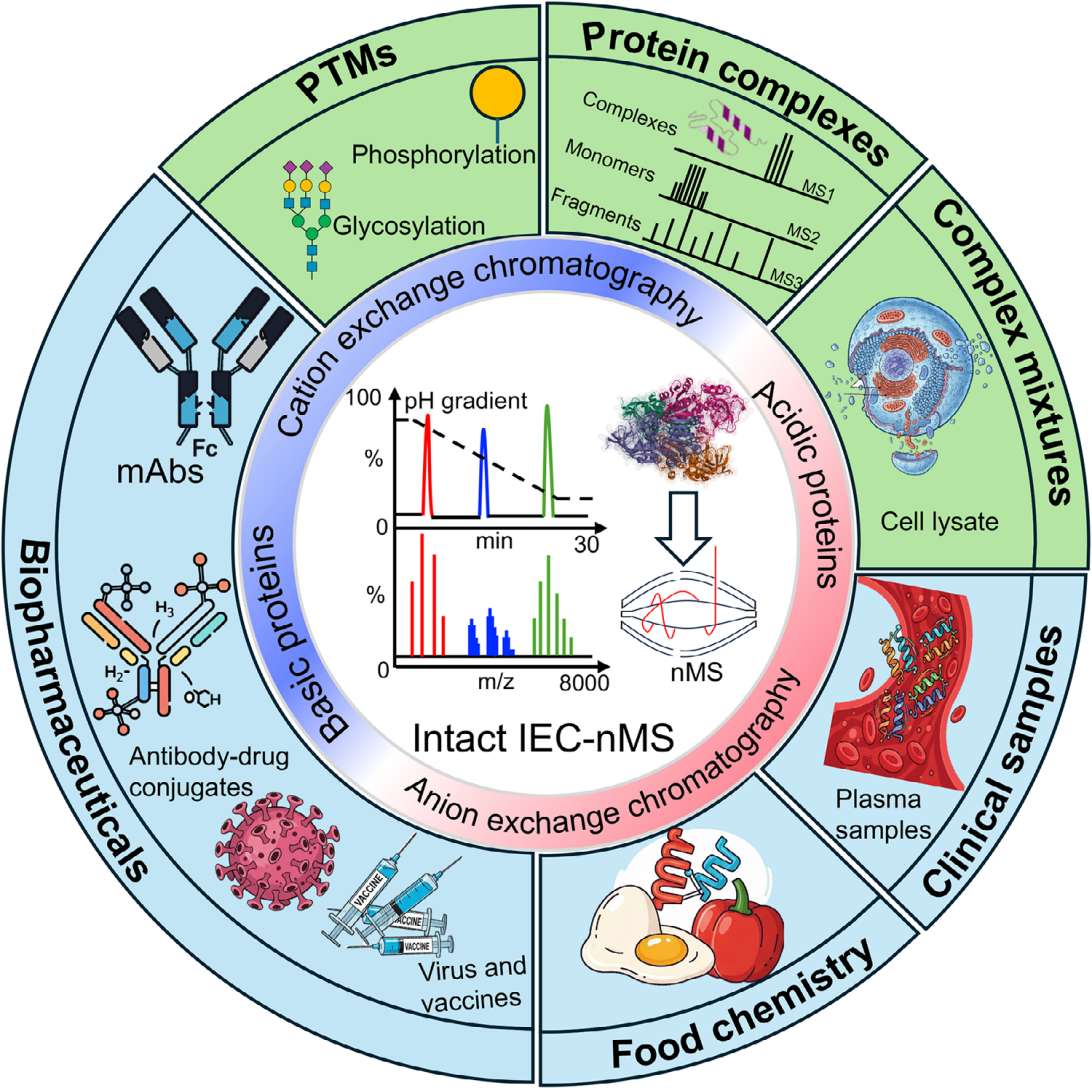
Overview of the applications (blue color) and multilevel information (green color) that intact IEC–native MS (nMS) can achieve.

Among these techniques, ion exchange chromatography (IEC) primarily separates proteins based on their net surface charge, and additional factors influencing the separation process will be discussed later [20]. Traditional IEC methods often require modifications for compatibility with MS [21]. Here, we describe recent developments and applications of IEC–MS for characterizing intact proteins and proteoforms under non‐denaturing or native conditions (Figure 1), based on research papers published between 2010 and early 2025. This review begins by outlining the fundamental principles of various IEC elution modes. Following this, we examine the critical influence of buffer system selection, column characteristics, and pH gradient design on protein separation efficiency. We then discuss strategies for the hyphenation of IEC with MS. Finally, we explore promising future directions in the analysis of native and complex samples, particularly within challenging matrices.

## 2 IEC Method Principles

### 2.1 Protein Retention in IEC and IEC Modes

**FIGURE 2:**
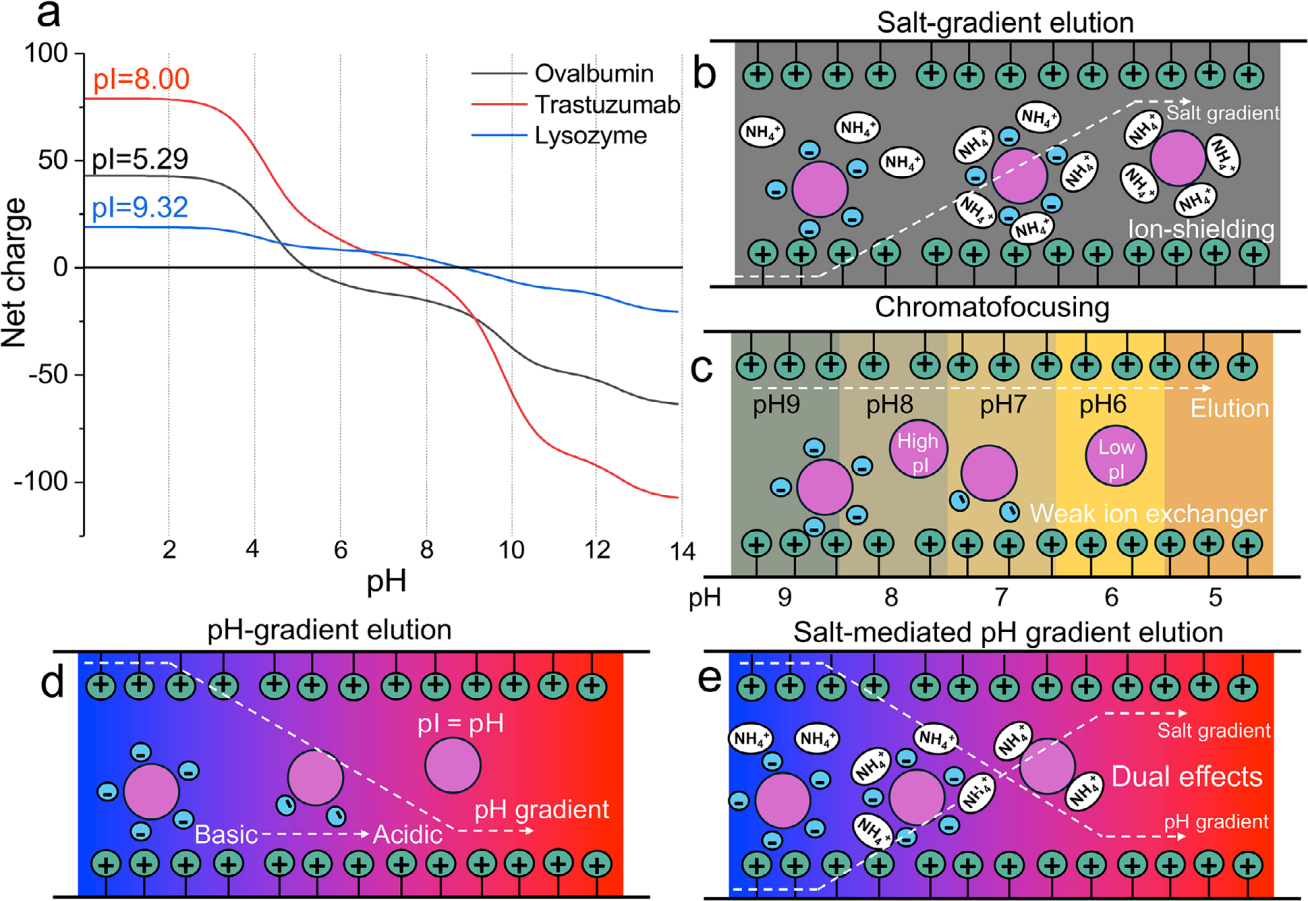
(a) Theoretical net charge changes of reference proteins. Illustration generated based on the protein sequences (P01012, P00698, HC, and LC of trastuzumab). (b–e) Illustration of time based (left initial, right final conditions) protein elution in anion exchange chromatography with salt gradient method (b), chromatofocusing (c), pH gradient method (d), and salt‐mediated pH gradient method (e).

Proteins are amphoteric molecules, possessing both positive and negative charges due to the presence of ionizable amino acid side chains and two terminal groups [22, 23]. The positive charge is usually attributed to the ionization of lysine and arginine side chains, N‐terminus, and histidine at lower pH values, while the negative charge is from the ionization of aspartate and glutamate side chains, and C‐terminus [24, 25]. The overall net charge of a protein is dependent on the pH of its surrounding environment. The pH at which a protein carries no net charge is defined as its isoelectric point (pI) [26, 27, 28]. Above its pI, a protein will have a net negative charge, whereas below its pI, it will have a net positive charge [29]. Therefore, in IEC, the pH of the mobile phase is selected to ensure that the net charge on the protein surface is opposite to that of the stationary phase (SPs). This charge difference allows the protein to displace the counterions associated with the functional groups of the SP, thereby binding to the chromatographic matrix [30, 31]. This property is fundamental to IEC, as it determines the interaction between the protein and the charged SP, enabling separation based on differences in protein net charges and pI values. As illustrated in Figure 2a for three model proteins (ovalbumin, trastuzumab, and lysozyme), the net charge of a protein varies significantly with the pH conditions. For example, to ensure retention of all three proteins illustrated on a cation exchange (CEX) column (negatively charged SP), the mobile phase pH should be approximately 4 or lower, ensuring a net positive charge on all proteins.

There are two main modes of IEC: anion exchange (AEX) and CEX. AEX uses a positively charged SP to separate negatively charged (acidic) species, while CEX employs a negatively charged SP to separate positively charged (basic) species (authors also use AEC and CEC to indicate AEX and CEX chromatography, however, in this manuscript the suffix X is used as abbreviation for exchange). Based on the chemical properties of the ion exchangers, AEX and CEX can be further classified into weak and strong exchangers. Weak cation exchange (WCX) SPs typically contain carboxylic acid groups, whereas strong cation exchange (SCX) phases often feature sulfonic acid groups. Similarly, weak anion exchange (WAX) phases may contain ternary amino groups, and strong anion exchange (SAX) phases usually have quaternary ammonium groups [32]. The weak exchangers will gradually lose their charges with changing pH, while the strong exchangers can maintain their charges over a wide pH range (e.g., between 2 and 12) [32]. The selection of the exchangers depends on the sample properties. However, it is often preferred to use strong exchangers for the method development as they allow for a wider range of pH [33].

### 2.2 IEC Elution Strategies

The retention of proteins in IEC is often described as an “on‐off” mechanism [34, 35]. This means that under conditions where the protein's charge is strongly attracted to the SP, binding is significant (“on”). Elution is typically achieved by changing the mobile phase (e.g., increasing ionic strength or changing pH) to disrupt these interactions, leading to a relatively rapid release of the bound proteins from the column (“off”). When considering the semi‐empirical linear solvent strength (LSS) model [36], such behavior will result in a very high S parameter value. Therefore, it is practically impossible to analyze large proteins under isocratic conditions, where even slight variations in conditions can lead to either no retention (solutes are entirely released from the SP) or complete adsorption (solute molecules are infinitely bound at the column inlet). Therefore, the application of gradient elution is essential.

Two main elution strategies are used in IEC methods: (i) salt gradient methods at constant pH, (ii) methods employing pH changes during the analysis (often accompanied by ionic strength changes).

i IEC coupled to UV detection (IEC–UV) or fluorescence detection (IEC–FLD) methods frequently apply gradients of ionic strength (Figure 2b) to separate charge variants of proteins [37]. In this case, the elution is primarily governed by the ionic strength of counter ions in the mobile phase. Gradients generally start with a low salt concentration (e.g., 20 mM) and increase to several hundred millimolar of a nonvolatile salt, such as sodium chloride [38].

The pI of the protein and pH of the mobile phase are critical factors that determine the specific salt composition and gradient steepness required for IEC analysis. To maintain a defined pH range, buffers such as phosphate or 2‐(*N*‐morpholino) methanesulfonic acid (MES) are usually employed, with acidic conditions (e.g., pH 5–6) favoring CEX chromatography and basic conditions (e.g., pH 8–9) favoring AEX chromatography [39, 40]. For example, in CEX, proteins and proteoforms are eluted based on their net charge, whereby species with a greater number of accessible positive charges require higher salt concentrations for the displacement from the SP.

While salt‐based gradients offer high separation power, they typically rely on nonvolatile additives and are therefore incompatible with MS. Although methods using volatile salts have been reported, the required high ionic strength (e.g., up to 500 mM) can lead to significant signal suppression and instability of the electrospray current, posing challenges for the MS hyphenation and sensitive MS detection.

ii pH‐based elution employs pH gradients to elute and separate proteins (Figure 2c–e). This is typically achieved using buffer systems and can sometimes be accompanied by ion strength gradients (e.g., low to high concentration of buffers or salts). Recently, these strategies have gained interest as alternative methods for profiling the charge heterogeneity of proteins, especially with the prospect of facilitating the hyphenation to MS.

pH gradients can be applied to characterize heterogeneous proteins, offering resolution comparable to or even exceeding that achieved with salt‐gradient methods [41]. In these methods, proteins are focused into narrow bands along the pH gradient. Moreover, pH gradients reduce the concentrations of volatile salts required during elution, a key factor contributing to the successful implementation of various IEC–MS methods [42, 43].

This review focuses on pH‐based IEC methods and describes three main approaches reported in literature: chromatofocusing, pH gradients, and salt‐mediated pH gradients (or pH‐assisted salt gradients).

### 2.3 pH‐Based Elution Strategies in IEC

There are two main ways to generate a pH gradient for IEC analysis. One method, known as chromatofocusing, also referred to as internal pH gradient, uses the buffering capacity of the SP itself to create a pH gradient that develops along the length of the column as elution proceeds [44]. The other approach, known as the external pH gradient, involves externally mixing two solutions with different pH values to generate a preformed pH gradient that is introduced at the column inlet [41, 45].

Chromatofocusing (Figure 2c) was one of the first forms of pH‐based elution in IEC. Although this term is sometimes used to indicate more general pH gradient approaches, here we refer specifically to this as the method first introduced by Sluyterman and Elgersma in 1978 [46, 47]. In chromatofocusing, a pH gradient is generated within the IEC column (typically a WAX column with suitable buffer capacity), equilibrating the column with a buffer at one pH (typically high pH) and then eluting with another buffer at a different pH (typically low pH) [44, 48]. The buffer capacity of the ion exchanger (SP) plays a crucial role in separating analytes during chromatofocusing. The chromatographic focusing effect arises as proteins migrate through the column and experience a changing pH environment. For example, consider a protein with a pI of 7, which is lower than the initial mobile phase pH (e.g., pH 9) and higher than the final pH (e.g., pH 6). Upon injection, the protein will be negatively charged and bind to a positively charged AEX SPs. As the pH decreases from high to low, it can equal the pI (pH = pI = 7) of proteins and even drop below it (e.g., pH 6), resulting in a net‐zero charge or positive charge and triggering protein elution. Proteins with different pI values will focus and elute at distinct points along the pH gradient. The steepness of the pH gradient influences the resulting width of the focused band. Chromatofocusing separations have been primarily achieved by employing ampholyte and nonvolatile buffers to establish a controlled pH gradient throughout the elution process [49, 50]. Although preparative and analytical IEC methods based on chromatofocusing have been described, the nonvolatile and ionic nature of their mobile phase components significantly limits their direct application in IEC–MS.

External pH gradient methods, involve changing the pH of the mobile phase over time (Figure 2d) [41, 45]. Proteins are retained based on their net charge at a specific pH, and elution is achieved by changing the pH within the column. The pH gradient is generated by externally mixing two or more buffer components, in the ideal case, producing a linear pH change during the analysis. The p*K*_a_ values of selected mobile phase components should fall within the desired pH range to provide sufficient buffer capacity and maintain a linear pH change profile. In addition, the apparent pH of the mobile phase can be significantly affected by the buffer capacity of the SPs [51]. Therefore, to obtain a linear pH change over time, the column and mobile phases are the primary factors that must be considered.

pH‐gradient methods can be performed at constant buffer ionic strength (hereafter referred to as pH gradients) or combined with an ionic strength gradient; the latter approach is referred to as salt‐mediated pH gradient methods (or pH‐assisted salt gradients; Figure 2e) [39, 42]. Salt‐mediated pH gradient method combines salt and pH gradient elution, providing additional elution control of proteins. It can overcome issues of buffer capacity and linearity of the pH change that occur in pH gradients over wide pH ranges (e.g., co‐elution resulting from insufficient buffering at certain pH values). This allows for extending the accessible pI separation range for complex samples with broad pI heterogeneity, even when relatively narrow pH ranges are used (e.g., 3 or 4 pH units). Overall, pH‐gradient strategies employing volatile salts are the most widely used approaches to achieve successful IEC–MS.

### 2.4 Factors Influencing Protein Elution in pH‐Mediated IEC

Although a common way to describe the elution mechanism of pH gradients is that proteins elute when the pH of the mobile phase reaches their pI values, where the protein carries no net charge, experimental observations often deviate from this idealized behavior. Studies have revealed differences between the elution pH and the expected pI values, ranging from less than one pH unit to several pH units above or below the anticipated pI [48, 52]. This discrepancy highlights the involvement of multiple factors beyond simple charge interactions that influence protein adsorption and elution in IEC. Regnier et al. proposed that the deviations presumably result from charge asymmetry because only a fraction of the protein surface interacts with the SP [53]. The protein retention and the number of charges associated with the adsorption‐desorption process presented a positive correlation.

Another key factor in protein retention is the ionic strength of the mobile phase, which affects the electrostatic interactions between proteins and the SP [54, 55]. Stern's theory explains how increased ionic strength reduces protein adsorption by the shielding effect of counterions, which weakens the electrostatic attraction between the protein and the charged surface [56]. Besides, the retention of proteins could also be affected by the types of displacing salts, SP ion exchangers, and is suggested to correlate with the ionic size, density, and thermodynamic activity [53]. An important consideration is the difference between the pH at the surface of the SP and the pH of the bulk of the mobile phase. The differences between these two pH values, known as the Donnan equilibrium effect, arise from the electrostatic interactions between charged groups on the SP and ions in the solution [56, 57]. In AEX, the surface pH tends to be higher than the bulk pH due to the repulsion of protons by the positively charged SP [56]. Conversely, in CEX, the surface pH is lower than the bulk pH. This surface pH effect can significantly alter the charge states of proteins near the SP, influencing their binding and elution behavior. Moreover, after the attraction effect between the ion exchanger and free proteins reaches a certain level, the repulsive charge effect between similarly charged analytes and the ions bonded on the SP will appear and have an extra influence on the protein adsorption and retention [58].

**FIGURE 3:**
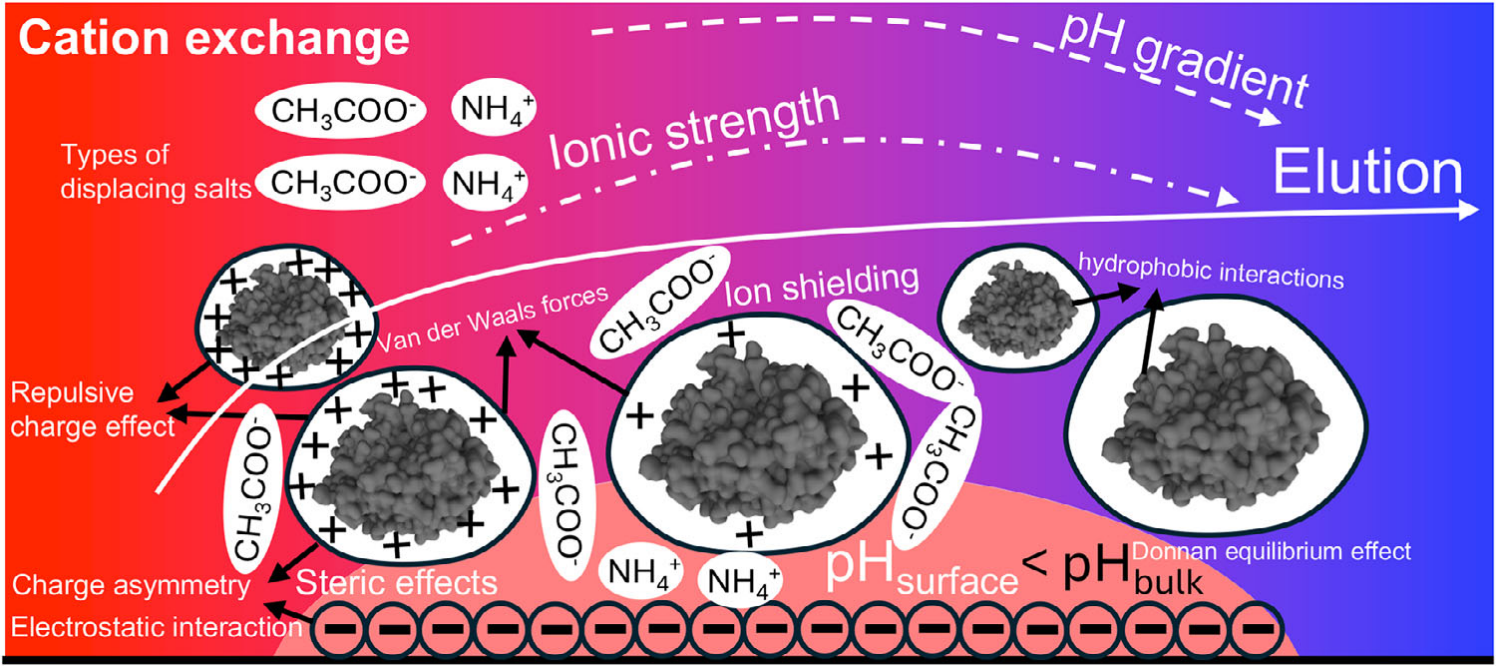
Illustration of factors that can affect the adsorption and elution of proteins in IEC (the cation exchange is taken as the example).

In addition to (i) charge asymmetry, (ii) ionic strength, (iii) types of displacing salts, (iv) surface pH effects, and (v) repulsive charge effect, various non‐electrostatic interactions contribute to the complexity of protein retention and elution in IEC (Figure 3) [59, 60, 61, 62]. These include (vi) steric effects, where the size and shape of the protein molecule can hinder its access to binding sites on the SP [63, 64, 65, 66], (vii) van der Waals forces, which involve weak attractive forces between molecules [67, 68, 69], and (viii) hydrophobic interactions, which occur between nonpolar regions of the protein and the SP [70, 71]. In salt‐mediated pH gradient elution, where both salt concentration and pH change simultaneously, the interplay of these factors becomes even more complex as the additional influence of the salt gradient can significantly alter the protein behavior.

As developed, mathematical models have been built and exploited to explain and simulate the ion exchange process. Early models of protein adsorption in IEC focused primarily on ion exchange, assuming a reversible stoichiometric displacement of ions on the SP by the protein [64, 72]. However, these models (e.g., stoichiometric displacement model) failed to account for the complex interplay of factors mentioned above. More recent models have incorporated steric effects, leading to the development of steric mass action models (SMA), which better describe the nonlinear adsorption behavior of proteins by controlling four critical parameters: characteristic charge, equilibrium coefficient, shielding factor, and kinetic coefficient [63, 73]. However, the assumption of thermodynamically ideal condition in SMA model is difficult to achieve. Raje and Pinto then proposed the SMA nonideal surface solution model (SMA‐NISS) [74], which includes specific and nonspecific adsorption effects in a thermodynamically consistent manner, such as ion exchange, steric effects, van der Waals, electrostatic interactions between adsorbed proteins and salts, and hydrophobic interactions between proteins and the adsorbate. In addition, the traditional SMA model believed that the protein is in a single or fixed charge state, impeding the simulation of the adsorption process. Shen and Frey extended the SMA model by incorporating the effect of charge regulation (three charge states of *z*, 0, and −*z*) of proteins to determine the conditions for an adsorbed species to displace a protein when the pH approaches the protein's pI [73].

Another limitation of SMA is the assumption of complete exclusion of co‐ions in the resin, which results in the pH value of the resin phase being the same as that of the mobile phase and the loading of counter‐ions being limited to the ionic capacity of the resin. As mentioned earlier, the observed Donnan equilibrium effect does not support this phenomenon. The Donnan ion exchange models (DIX) were then created to characterize the resin phase pH and ion concentrations [75, 76]. Sometimes, it is challenging to obtain detailed information on the analytes and the structure of the SP for predicting protein retention. Yamamoto et al. provided a simple model [77, 78, 79], the linear gradient elution model (LGE), to predict the peak position and peak width by only focusing on the gradient slope, flow velocity, and column length. In addition, the observed discrepancies between experimental data and model results indicated that the stoichiometric models (e.g., SMA) could not provide an accurate description of nonlinear adsorption behavior. Given the colloidal nature of proteins, a colloidal particle adsorption model (CPA) was proposed by Briskot et al. to depict the protein adsorption as a function of ionic strength and pH, in which the nonlinear adsorption behavior is described as a combination of steric surface blocking effects and electrostatic interactions between adsorbed proteins, showing a better model results compared with SMA [71, 80]. Other models, such as general rate models [61, 81] and dispersive models [82, 83], have been developed to model the (macroscopic) mass transfer through the column.

Overall, the traditional concept of protein adsorption and elution at the point of pH = pI is not sufficient to explain the protein behavior in IEC. Apart from the ionic strength of mobile phases and surface charges of proteins, the other micro‐interactions also play essential roles during the elution process. Moreover, many different types of related models have been created to predict the protein elution, which helps understand how the diverse factors work during this process.

## 3 Recent Trends in IEC–MS Methods

### 3.1 Mobile Phases for IEC–MS: Buffer Systems and pH Linearity

**FIGURE 4:**
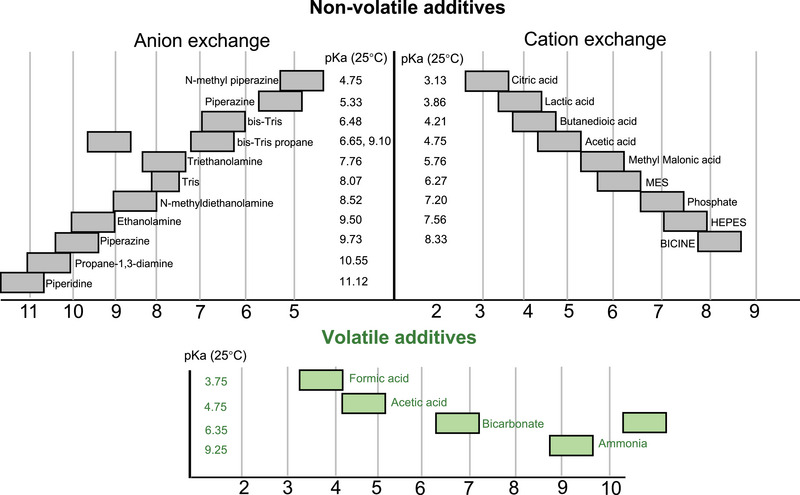
The overview of various nonvolatile and volatile additives used in CEX and AEX systems, adapted from [88]. The gray color means the nonvolatile additives and green color represent the available volatile buffer systems.

Direct IEC–MS methods rely on the use of volatile buffer components (e.g., ammonium acetate [AmAc]) to enable IEC separation while maintaining MS compatibility [84, 85]. For the IEC–UV, a range of nonvolatile salts with p*K*_a_ values spanning from 3 to 10 can be selected for CEX or AEX, allowing pH gradients running over a broad range (Figure 4). In contrast, when performing IEC–MS, only a limited number of volatile additives are available: ammonia, acetic acid (AA), formic acid (FA), ammonium carbonate (AmCa), ammonium bicarbonate (AmBc), AmAc, ammonium formate (AmFo).

AmAc and AmFo are the most commonly employed systems. AmAc is a kosmotropic salt and can stabilize protein structures, whereas AmFo is a chaotropic salt and can disrupt hydrophobic interactions in proteins [86]. It should be noted that AmCa/AmBc has been reported to denature proteins during native MS analysis, yielding spectra with high charge‐state distributions. This effect has been attributed to CO_2_ outgassing during heating of protein‐containing bicarbonate solutions [87].

Despite its advantages, direct IEC–MS using volatile salts often presents challenges compared to traditional methods employing nonvolatile additives. The peak shape and symmetry achieved with volatile salts are generally inferior to those obtained with conventional nonvolatile buffers, such as MES and 4‐2‐hydroxyethyl‐1‐piperazineethanesulfonic acid (HEPES) systems [89, 90, 91, 92]. Furthermore, volatile salts typically exhibit lower buffer capacity and ionic strength than their nonvolatile counterparts, and therefore, high concentrations of salts may be needed in salt‐mediated pH gradients (e.g., > 200 mM). High salt concentrations can be problematic in ESI ionization, potentially resulting in reduced sensitivity due to ion suppression effects, insufficient desolvation, and adduct formation in the mass spectrometer [93, 94, 95].

While pH‐gradient IEC mitigates the need for high‐ionic strength mobile phases to achieve high‐resolution protein separation, the limited availability of volatile buffers can impair the ability of mobile phases to effectively control pH, making it challenging to obtain the linear pH change required for accurate separation of charge variants, especially those with very similar properties. In general, uncontrolled pH changes in gradient methods can lead to abrupt, or non‐proportional shifts in pH (e.g., several pH units changing in a short time) due to insufficient buffer capacity within the required pH range [96]. This increases the risk of co‐elution for analytes with closely related pI values within narrow elution windows. Therefore, careful control of the pH change per unit of time is crucial, and the inherent buffering capacity of the IEC column itself, which can influence the pH gradient profile, must be considered [51]. Volatile buffers compatible with MS detection are limited to ammonia (from ammonium hydroxide [AmHy] solution)/FA, ammonia/AA, and ammonia/AmBc. These systems can effectively buffer only within limited pH ranges (Figure 4): 3.3–4.3 (FA), 4.3–5.3 (AA), and 8.8–9.8 (ammonia). Controlling a linear pH gradient between 5.3 and 8.8 with suitable volatile additives remains challenging, as ammonia/AmBc has several drawbacks (e.g., protein denaturation during ESI) and is often not preferred. In addition, it is worth pointing out that when using these mobile phases with glass bottles, laboratory glassware, or glass vials, there can be a loss of MS resolution, sensitivity, and mass accuracy due to the introduction of metal contaminants. Therefore, it is advisable to avoid using them during analysis [97].

Achieving a linear pH response with volatile additives remains a key challenge in IEC–MS analysis. To address this, three main strategies can be explored: (i) implementing new volatile additives that can extend the buffer ranges, (ii) correcting nonlinear pH profiles by programming the HPLC pump gradient, (iii) implementing salt‐assisted elution and therefore narrowing the pH gradient window where a linear pH change is required.

**FIGURE 5:**
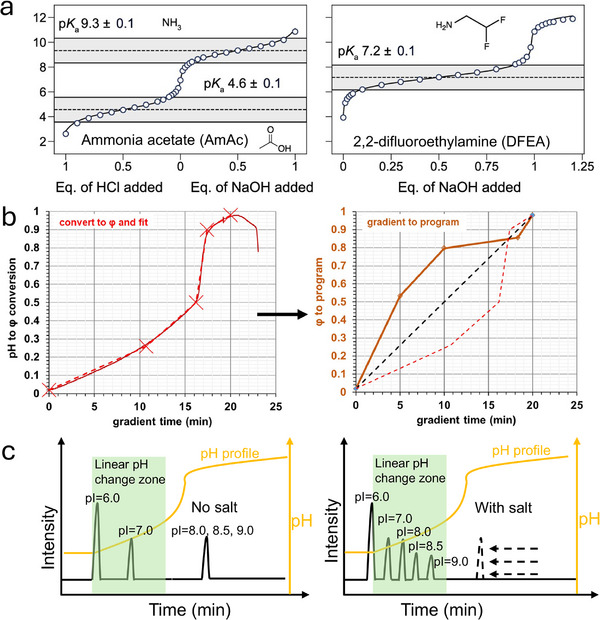
Strategies to obtain the linear pH response with volatile additives in the IEC–MS analysis. (a) Potentiometric pH titration of AmAc and novel volatile salt of DFEA. Figure adapted from Davis et al. [98] with permission from American Chemical Society. (b) Illustration of the correction of nonlinear pH profile method (red dotted trace represents the function of the original pH gradient, and the dark red solid trace represents the inverse function of the original pH gradient). Figure adapted from Fekete et al. [100] with permission from Elsevier. (c) Illustration of the salt‐assisted elution and its advantage in facilitating the elution of charge variants within the linear pH area of the gradient.

- One promising approach involves the use of electrospray‐compatible buffers with p*K*_a_ values that fill the existing gaps in buffer availability. In this context, Davis et al. recently introduced 2,2‐difluoroethylamine (DFEA) and 2,2,2‐trifluoroethylamine (TFEA) (Figure 5a,b) [98]. DFEA exhibits a p*K*_a_ of 7.2 with a buffer capacity spanning approximately pH 6.1–8.1, while TFEA has a p*K*_a_ of 5.5 and buffers effectively from around pH 4.2–6.2. For the use in IEC, it has to be considered that TFEA and DFEA are fluorinated reagents and may act additionally as ion pairing agents, increasing retention of proteins with hydrophobic surfaces. Besides, Hadavi et al. developed a buffer of 4‐ethylmorpholinium/acetate with p*K*_a_ of 7.72/4.76 for native MS, which can lead to lower charge states compared with AmAc and preserve protein folding during nano‐ESI [99]. These novel additives show significant potential to bridge the current buffering gap in the pH range of 5–8. Notably, a combination of FA, AA, AmFo, AmAc, DFEA, TFEA, and AmHy has demonstrated the ability to buffer a broad pH range from 3.6 to 10.3. However, it is important to note that these two novel volatile salts have been tested to date only using static nanospray ESI. Further research is needed to evaluate their behavior in IEC mobile phases and their influence on separation performance.
- Another strategy focuses on correcting the nonlinear pH profiles that can arise with existing volatile buffers. Fekete et al. developed a novel approach (Figure 5b) for this purpose [100]. This method involves first recording the actual pH profile (pH vs. time) of the gradient method using an online pH meter. Subsequently, the pH data is converted to mobile phase fraction, and an inverse response function is generated in the time domain (mobile phase fraction vs. time). This inverse function is then utilized as the gradient program to actively correct the original nonlinear pH response, resulting in a more linear pH profile. This approach provides a relatively simple and rapid method for enhancing pH linearity, considering the column's buffering capacity. However, it may have limitations in correcting for very rapid pH changes or large pH shifts occurring over short periods (e.g., 2 pH units).
- The third strategy is salt‐mediated pH gradient method. It leverages the ion shielding effect or the displacement principle to achieve earlier elution of proteins, thereby enabling the separation to occur within a known linear pH response zone of the pH gradient (Figure 5c). For example, Zhai et al. developed a salt‐mediated pH gradient method with a linear pH change window from pH 5 to 6.5, spanning approximately 8–26 min. In this method, up to 250 mM AmAc was employed to facilitate the rapid elution of proteins, ensuring that the separation took place within the identified linear pH response window [20]. However, the determination of the optimal linear pH change window and the appropriate concentrations of salts in such salt‐mediated pH gradient methods requires careful investigation and optimization.

### 3.2 SPs and Columns for IEC–MS

This section describes some of the critical aspects of IEC column technology for protein separation, discussing, in particular, (i) the type of SP materials used in IEC–MS and (ii) considerations for column selection.

- Polymer‐functionalized ion exchangers are the most popular materials for IEC because of their characteristics such as high pH stability, high charge density (selector coverage), and fast mass transfer rates (nonporous materials) [32, 101, 102, 103]. The SPs are usually composed of a support polymer (substrate) and coupled ion exchange groups. The amount and nature of the ion exchange group determines the charge density (influencing the binding strength of proteins). The physical and chemical properties of support determine the attainable number of ion exchange groups, stability, and flow characteristics of the ion exchanger [104].

Currently, the most widely used polymers are styrene or ethylvinylbenzene (EVB) (sometimes used in mixtures) copolymers cross‐linked with divinylbenzene (DVB) [105, 106], although poly(vinylalcohol) [107] and poly(methacrylates) [108, 109] have also been described and are present in commercial products (e.g., Sartorius, BIA Separations).

Polystyrene (PS)–DVB and EVB–DVB excel in IEC due to their exceptional pH resistance, which allows for operation without degradation across a wide pH range from 0 to 14, as well as high temperature and mechanical stability. To introduce ion exchange functionality, these DVB‐based polymeric backbones are chemically functionalized. For CEX materials, this typically involves sulfonation, where sulfonic acid groups are directly attached to the aromatic rings of the polymer, or carboxylation, where carboxylic acid groups are attached. For AEX materials, the polymer is usually subjected to chloromethylation, followed by quaternization of tertiary amines to introduce positively charged functional groups, such as quaternary ammonium groups.

However, the hydrophobic nature of PS–DVB and EVB–DVB polymers can induce protein denaturation due to specific non‐electrostatic interactions, such as van der Waals forces and π–π interactions, between ionized analytes and the SP [110]. To mitigate this, polymer SPs that integrate hydrophilic polymers through co‐polymerization or grafting have been studied. Arrua et al. proposed that the monolithic column fabricated by copolymerizing hydrophilic monomers and crosslinkers is a direct way to obtain with reduced secondary interactions [111]. In addition, a hydrophilic layer can be grafted to reduce nonspecific hydrophobic interactions [110, 112, 113, 114].

Recently, some related monolithic IEC materials have also been described and used in some instances, such as the copolymerization of phosphoric acid 2‐hydroxyethyl methacrylate (PAHEMA) and polyethylene glycol diacrylate (PEGDA), or bis[2‐(methacryloyloxy)ethyl] phosphate (BMEP) and polyethylene glycol acrylate (PEGA) [115]. Arrua et al. summarized the recent advances in organic polymer monoliths developed for the IEC of large molecules [111].

In terms of SP morphology, nonporous IEC SPs are typically employed in IEC methods for protein analysis and were used in the majority of the studies reported in this review (49 over 53 manuscripts analyzed in this review). Their high charge density allows for increased binding capacity and sample loading, while the SP morphology reduces mass transfer‐driven band broadening, which is especially critical due to the low diffusion coefficients of large proteins [32, 116]. SP with diameters of 3, 5, and 10 µm are frequently used in the commercially available IEC columns. As the particle size affects the specific surface area, Murisier et al. found that the columns with larger particle sizes showed lower retention, while the relationship between efficiency and particle size was not observed [117]. Furthermore, another study suggested that further reducing IEC particles below 2.5 µm may lead to more drawbacks than benefits [116].

ii In terms of column format, wide bore IEC columns (4–4.6 mm ID) are commonly used for routine separations at the flow rate of 0.6–1.2 mL/min and are especially suitable for UV/FLD based studies. In contrast, the narrow bore columns (2‐ or 2.1‐mm ID) may be preferred for MS coupling as they allow using lower flow rate (0.1–0.6 mL/min), facilitating ESI coupling [33, 117, 118]. Some studies have proposed to use the narrower capillary IEC columns with a diameter of 200 µm to further reduce the flow rate to a nanoscale level, such as 0.5 µL/min [20]. The lower flow rate is more suitable for online MS detection, with increased sensitivity due to the higher desolvation efficiency [119, 120].

Several vendors offer IEC columns and most of them have been used in IEC–MS studies. The performance of state‐of‐the‐art IEC columns for protein separation has been summarized in the review by Fekete et al. [32] (previously discussed also by Staby et al. investigating IEC–UV performance [121, 122, 123, 124, 125, 126]). In particular, Fekete et al. compared the retention, selectivity, and resolving power for characterization of mAbs charge variants using both pH gradient and salt gradient with five different CEX columns: Sepax Antibodix WCX‐NP3 (particle size 3 µm × column length 15 cm), Thermo MAbPac SCX‐10 RS (particle size 5 µm × column length 15 cm), YMC BioPro SP‐F (particle size 5 µm × column length 10 cm), Waters Protein‐Pak Hi Res SP (particle size 7 µm × column length 10 cm), and Agilent Bio mAb NP1.7 SS (particle size 1.7 µm × column length 5 cm) [33]. They found that YMC Bio Pro SP‐F material is less retentive compared with Bio mAb NP1.7 SS (which gave the highest retention) but has the highest resolution in most cases. The MabPac SCX column can give higher selectivity for the main mAbs isoforms and variants. Similarly, Murisier et al. reported that different combinations of SP and mobile phases can result in diverse retention, selectivity, and efficiency in the analysis of therapeutic mAbs [117].

Finally, it is worth pointing out that mixed‐bed‐IEC, first described in the 1980/1990s by coupling AEX and CEX columns in series or creating mixed beds, have recently been implemented using mixtures of wide‐pore silica‐based IEC materials to achieve retention and separation of complex protein mixtures [127, 128, 129, 130], including both acidic and basic proteins and proteoforms under MS‐compatible conditions [131, 132].

### 3.3 Approaches for Coupling IEC to MS

Both (i) indirect and (ii) direct approaches for coupling IEC with MS have been described and recently applied.

- For specific applications, particularly where IEC–UV methods based on nonvolatile buffers are already well‐established (e.g., mAb analysis), MS detection serves as a confirmatory tool. In such cases, indirect coupling strategies, which utilize either volatile or nonvolatile buffer systems in the IEC mobile phase, are a common choice preferred to redevelop an IEC method using volatile buffers [133, 134].

**FIGURE 6:**
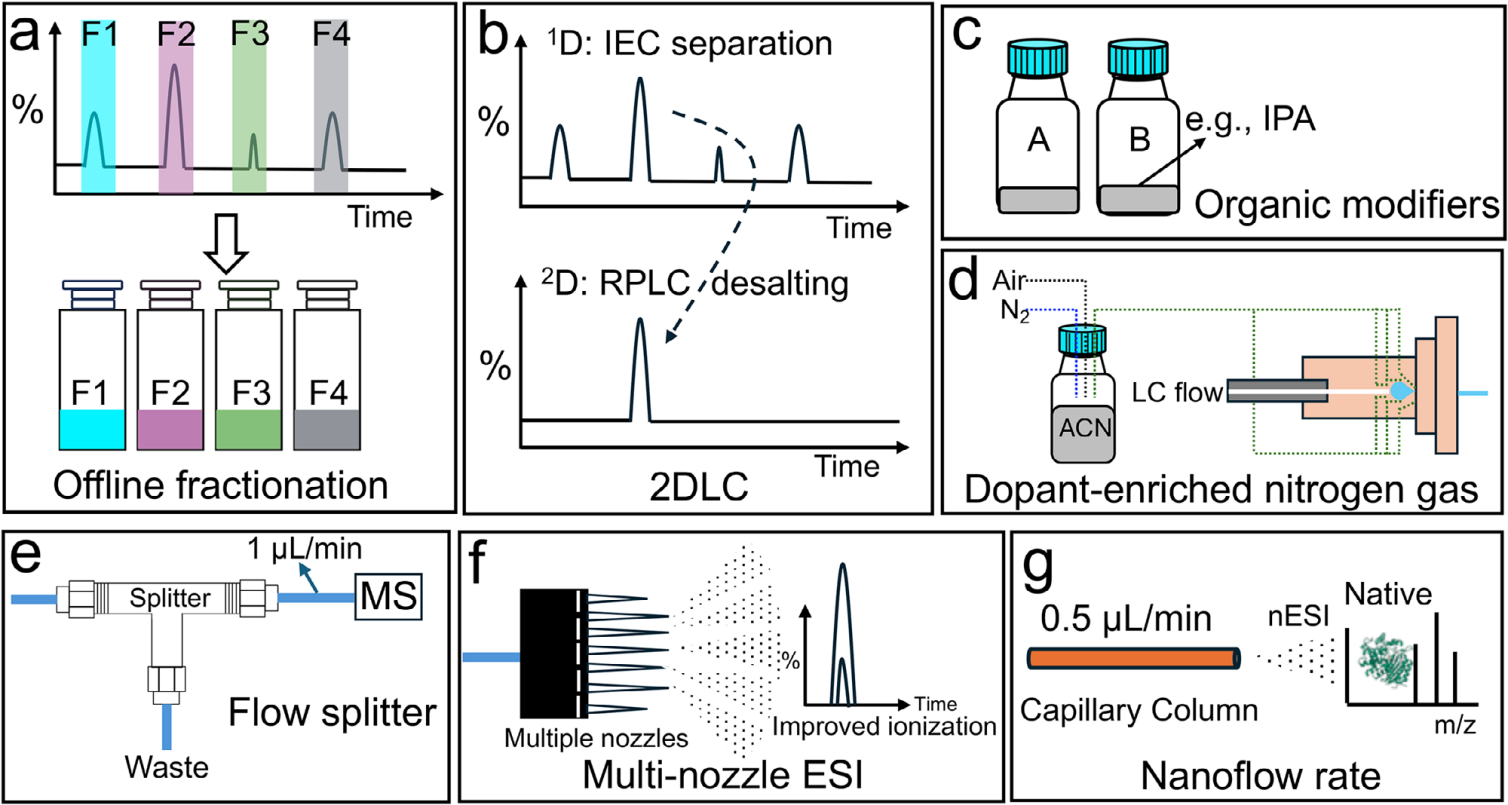
Illustration of diverse approaches to achieve IEC coupling to MS. (a) Offline fractionation, (b) 2DLC–MS, (c) organic modifiers, (d) dopant‐enriched nitrogen gas, (e) post‐column flow splitter, (f) multi‐nozzle ESI, (g) nano‐flow IEC–MS. Offline fractionation (a) and 2DLC (b) are indirect ways to achieve the IEC–MS. The other methods (c–g) are direct ways to achieve the IEC–MS. The (c) and (d) are strategies that use the addition of organic modifiers to facilitate MS coupling. The (e)–(g) are approaches that reduce the flow rate before ESI.

Indirect coupling can be generally achieved through offline fractionation followed by reinjection onto another MS‐compatible LC separation or desalting using membrane spin filters before static nano‐spray infusion (Figure 6a) [135]. For example, Baudhuin et al. collected fraction (Tris‐HCl/NaCl solvents) of antibodies separated by AEX chromatography and buffer exchanged them with membrane‐based spin filters before ESI–Q–ToF–MS analysis [136]. Another method to perform indirect IEC–MS coupling makes use of two‐dimensional LC (2DLC) to perform online buffer exchange of IEC (Figure 6b) [137, 138, 139, 140]. For example, Wu et al. utilized SAX (^1^D) to firstly separate adeno‐associated viruses (AAVs) by employing nonvolatile mobile phases composed of bis‐tris propane/tetramethylammonium chloride, and the interested peaks are then subjected to online desalting in a trap column followed by RPLC–MS [137].

However, indirect approaches can relatively increase analysis time, potentially altering the original sample states (e.g., inducing denaturation), and often introduce instrumental complexity (e.g., valves and fraction collection units). Such methods also carry risks of sample losses (e.g., during offline fractionation), sample dilution (e.g., when two chromatographic processes are combined in 2DLC), and deterioration of the initial IEC separation (e.g., band broadening during transfer or storage). Desalting procedures, though necessary to ensure MS compatibility, can exacerbate these drawbacks by risking protein denaturation, introducing artifact modifications, increasing analysis time, causing loss of low‐abundance species, and reducing overall throughput.

ii For the development of new methods or in cases where a comprehensive IEC–MS approach is needed, direct IEC–MS is typically the method of choice. However, directly coupling IEC separations that rely on high concentrations of water‐based buffer solutions presents a significant challenge for sensitive MS analysis. Direct IEC–MS methods have been only recently introduced with one of the first studies described in 2013 [141], later followed up in 2015–2018 [94, 142]. Since then, more and more implementation and approaches to realize this coupling have been reported.

Below are summarized some of the recent strategies described to facilitate the direct coupling of IEC to MS. In particular, (i) the addition of organic modifiers (during the separation or after the separation), (ii) approaches to reduce the flow rate to the MS.

- Organic modifiers, such as acetonitrile (ACN), enhance ionization in ESI–MS primarily by lowering the surface tension and increasing the volatility of the electrospray droplets. These effects promote the generation of smaller and highly charged droplets, which leads to highly efficient solvent evaporation and more effective ion release into the gas phase [97, 143].

Organic solvents, like ACN, can be added to the mobile phase, usually with less than 20%, and this has been described to enhance ionization in IEC–MS (Figure 6c). For instance, Dai et al. added 20% ACN into the AmAc‐based mobile phases for SCX–MS [144]. The addition of organic solvents can affect and, in some case, improve the IEC separation by affecting the hydrate shell between proteins [145]. Duivelshof et al. investigated a series of organic solvents and found that acetone should be avoided as it results in no peak elution [145]. The addition of the most organic solvents (isopropyl alcohol [IPA], dioxane, ethanol [EtOH], ACN, and dimethyl sulfoxide [DMSO]) in the mobile phases can lead to the later retention times, especially for ACN. The addition of butanol has been reported to have less influence on retention behaviors.

Organic solvents can also be introduced post‐column via T‐pieces or during ionization using dopant‐enhanced nitrogen (DEN) gas (Figure 6d). Under these conditions, flows/doped gas based on water‐ACN with FA as an additive are often applied to reduce pH, leading to protein denaturation and enhancing ESI ionization [146]. For example, Shi et al. [146] reported an almost 10 times increase in MS intensity in the SCX–MS analysis of a model mAb when mixing post‐column of 70% IPA, 5% H_2_O, and 25% FA at 110°C to facilitate unfolding (MS intensity 5.5E4 with 24+ to 30+ charge states for native vs 2.8E5 with average charge state of 41+). The authors also commented that a 10% addition of organic solvent in the MP did not lead to signal enhancement. In comparison, the addition of 20% organic solvents resulted in the appearance of additional CEX peaks, indicating that partial in‐solution or on‐column unfolding was taking place, leading to separation artefacts.

An alternative to the addition of flow after separation is the use of DEN gas ionization. This is achieved by enriching the nitrogen‐containing nebulizing gas with the solvent. Then the gas is introduced in the ion source around the spray emitter to assist the desolvation and ionization [147, 148, 149]. This approach has been described to further improve ionization efficiency in IEC–MS by promoting desolvation [150]. Studies have shown the effectiveness of DEN–MS in enhancing sensitivity for intact protein analysis, particularly when coupled with salt‐mediated pH gradients [151, 152, 153]. Furthermore, the DEN gas is proven to reduce adduct formation, particularly for the ACN DEN, and reduce charge states with respect to ESI, regardless of the selected dopants [150]. However, DEN ionization can only be applied at the low microliter flow rate and therefore its application to the analytical flow rate often involves the use of flow splitters before ionization.

ii Reducing the flow rate before ESI ionization is done in most of the native IEC–MS studies using analytical flow (e.g., 0.2 mL/min). The lower flow rate (e.g., below 20 µL/min) reduces the volume of water that needs to be evaporated before ESI–MS, facilitating the use of native ESI–MS conditions [15]. To achieve the lower flow rate, microflow LC (typically with the flow rate ranging from 1 to 100 µL/min) and post‐column flow splitting (often requiring a split ratio greater than 50‐fold) are frequently employed (Figure 6e) [154]. The advantage of microflow LC coupled with MS is the improved sensitivity, reduced solvent consumption, decreased matrix effects, and lower source contamination [155]. However, compared with the analytical flow regime, it often requires dedicated hardware for mobile phase delivery, fluidic connections, sample injection, chromatographic columns, and ionization sources [156]. Flow splitting is a relatively direct and simple way to achieve a lower flow rate. For example, Yan et al. reduced the flow from 0.4 mL/min to 1 µL/min in the SCX–nMS method with a post‐column splitter (≈ 400:1) to achieve sensitive analysis of charge variants of mAbs using salt‐mediated pH gradient elution [157]. Sensitivity losses are generally present with such a high flow splitting ratio, given that most of the sample is not directed toward the MS.

To overcome this issue, recent work has demonstrated the advantages of using multiple ESI nozzles to facilitate ionization and increase sensitivity (Figure 6f). Microfabricated monolithic multi‐nozzle (M3) divides the eluent flow into multiple electrospray emitters (e.g., 8). This allows a lower flow rate in each emitter, resulting in smaller droplets before ionization, leading to more efficient desolvation and ionization of the analytes [158]. A commercialized M3 solution exists, which cannot be directly used with analytical flow rates and requires a flow splitter to reduce the flow to typically 20 µL/min in the case of native LC conditions. Liu and Yan both employed the M3‐based IEC–MS to achieve a highly sensitive charge heterogeneity characterization of mAbs (sensitivity increased up to 50 times with respect to only flow splitting) [151, 152].

Finally, an alternative to using multi‐nozzle spray is to directly develop nano‐flow IEC–MS methods (Figure 6g), using capillary columns (currently these columns are lab‐made and are not yet commercially available) and a volatile salt‐mediated pH gradient [20]. It has higher requirements compared with microflow scale analysis and presents more excellent performance for intact protein analysis. This approach eliminates the need for heated gas and high voltages during the ionization, providing milder conditions for native protein analysis. Zhai et al. demonstrated the feasibility of this approach using refence proteins, for example, obtaining clear signals from mAbs with injections as low as 33 ng, compared with the tens of micrograms required in analytical‐flow experiments, and achieving high‐resolution separations that enabled the detection of hundreds of proteoforms, with masses up to 140 kDa, in a complex cell lysate [20].

## 4 Charge Variants Analysis by IEC–MS

IEC methods are highly effective in separating proteins based on their surface charge, enabling the resolution of distinct protein sequences and proteoforms characterized by charge variants. Charge variants can have different biological functions, depending on the location, type, and extent of the modification, and are therefore important to be characterized in protein products such as biopharmaceuticals [159, 160]. This section describes common proteoforms, categorizing them as either acidic or basic based on their charge properties. Acidic proteoforms typically arise from modifications that introduce negative charges or neutralize positive charges, examples include sialylation, phosphorylation, deamidation, C‐terminal lysine cleavage and acetylation.

Glycosylation refers to the product of an enzymatic process where a glycan is attached to an amino acid and can be divided into *N*‐ or *O*‐linked glycosylation. *O*‐Glycosylation usually bears oligosaccharides (e.g., *N*‐acetyl galactosamine, GalNAc) at a serine (Ser) or threonine (Thr) residue, while *N*‐glycosylation happens on an Asn‐Ser/Thr sequence with a common core structure of Man3GlcNAc2 [191, 192]. Neutral glycans do not introduce charge groups in the protein, but the presence, absence, and specific structures of them can induce conformational changes in the protein, inducing changes in the exposed charges. Glycan modification, such as the sulfation, does instead introduce charge variants. Similarly, sialylation, which arises from the addition of sialic acid units to the end of an oligosaccharide chain in *N*‐ or *O*‐linked glycoproteins, can also induce charge variants [193, 194]. The *N*‐acetylneuraminic acid (Neu5Ac) and *N*‐glycolylneuraminic acid (Neu5Gc) are the most common forms of sialic acids added to proteins. Compared to glycosylation, glycation arises from the nonenzymatic covalent attachment of reducing sugars (e.g., glucose) to the basic residues (e.g., lysine) of a protein, generally resulting in the addition of 162 Da in the case of hexose sugars [195]. The reaction neutralizes the positively charged residue groups of basic amino acids, thereby changing the charge of the protein. As the glycation reaction progresses, more complicated variants may be formed, including the advanced glycation end‐products.

Phosphorylation is the addition of a phosphoric acid group to proteins resulting in a mass increase of 80 Da. It usually happens on Ser/Thr, Tyr residues [196]. Deamidation results from the modification of the amide functional group of asparagine (Asn) or glutamine (Gln), which is exchanged for a carboxylic acid, typically leading to a 1 Da mass increase [196]. Acetylation refers to the covalent attachment of an acetyl group to either the α‐amino group of the N‐terminus of proteins or to the ε‐amino group of lysine residues, removing a positively charged group and resulting in a mass increase of 42 Da [197]. Acetylation can influence the lifespan, folding characteristics, and binding properties of proteins [174]. Amino acid truncation can be present in recombinant protein production, and if acidic or basic amino acids are removed, it can generate charge variants. In particular, C‐terminal lysine truncation is a common modification observed in mAbs, referring to the enzymatic or nonenzymatic removal of a C‐terminal lysine residue from the heavy chain, which results in a characteristic mass difference of 127 Da (mass of lysine) [198]. Glucuronylation results from the covalent attachment of a glucuronic acid to the hydroxy group of amino acid residues, mediated by uridine diphosphoglucuronosyl transferase (UGT) [199, 200, 201]. Finally, succinimidation is a succinimide intermediate, happening on asparagine or aspartic acid residue with a mass decrease of 17 Da (or −18 Da) [185, 202, 203].

Conversely, basic proteoforms may result from modifications that introduce positive charges or neutralize negative charges, such as C‐terminal amidation, N‐terminal glutamine cyclization (to pyroglutamate (pE), potentially affecting charge based on the original N‐terminus), and aglycosylation when charged glycans are lost. Moreover, conformational changes can also induce the formation of basic variants. The methionine oxidation is a common modification that increases mass of +16 Da [204, 205, 206]. In C‐terminal amidation, the opposite outcome of a deamidation is observed with the free carboxyl group (─COOH) at the C‐terminus of a protein or peptide being converted into a neutral amide group, removing a negatively charged amino acid in the protein and resulting in a 1 Da difference.

The formation of pE from the N‐terminal Glu or Gln may lead to acidic or basic variants, respectively, with the charge variant containing the pE being less basic than the one containing the Gln, and less acidic than the one containing the Glu [97, 207]. Aglycosylation refers to the absence or loss of glycosylation in a protein by introducing the mutation at the glycosylation site [180]. It can address the drawbacks of glycan heterogeneity which results in high variation of biological activity. Isomerization of aspartyl (Asp) residues is a common degradation way that leads to the variants of mAbs. It undergoes two steps (formation of succinimide intermediate and hydrolysis) to form the isoaspartate (isoAsp) [208].

Some proteoforms may result in either basic or acid variants. This is the case of protein cysteinylation, in which a cysteine residue on a protein forms a disulfide bond with a free cysteine molecule. In mAbs this happens in the hinge region peptide that involves four closely spaced cysteine residues of the heavy chain [185, 209]. Finally, aggregation/oligomerization is also considered one of the PTMs that can induce acid or basic variants, as in the formation of oligomers (e.g., dimer), charged amino acids are involved and therefore are not accessible on the protein surface [210].

Tables 2, 3, 4 summarize recent publications covered in this review (from 2010 to 2025), highlighting the samples and properties studied, as well as the characteristics of the methods applied in separation and MS. Most studies reported here describe the application of these methods for biopharmaceutical characterization (48/53), with a particular focus on mAb‐based pharmaceuticals (27 over 48), demonstrating the relevance and increasing interest (33 studies have been reported in the last 5 years) in IEC–MS for biopharmaceutical characterization.

**FIGURE 7:**
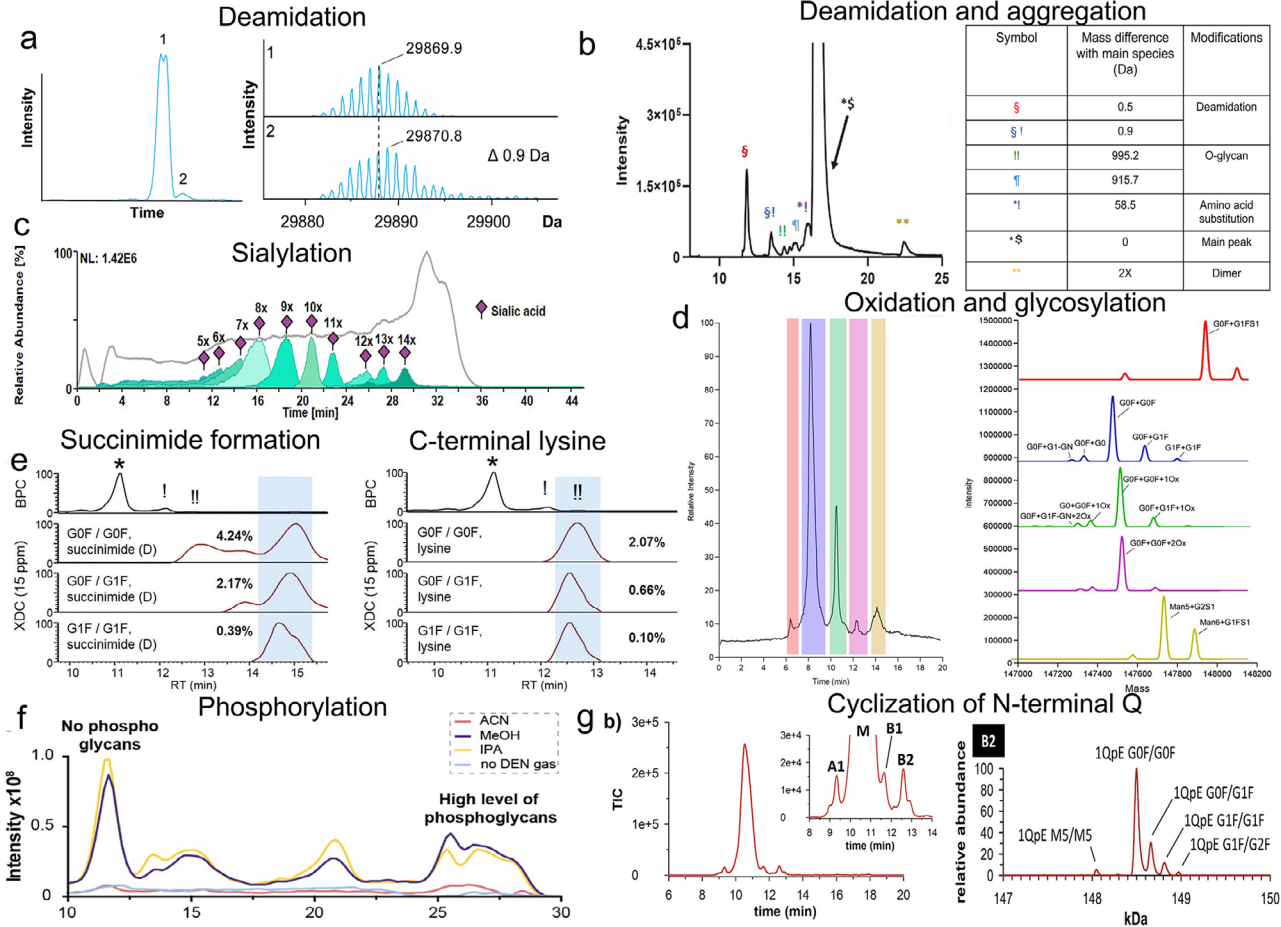
Examples of investigation for different types of modifications. (a) Deamidation. Figure adapted from van Schaick et al. [241] with permission from Elsevier. (b) Deamidation and aggregation. Figure adapted from Shah et al. [239] with permission from Elsevier. (c) Sialylation. Figure adapted from Di Marco et al. [242] with permission from Elsevier. (d) Oxidation and glycosylation. Figure adapted from Lambiase et al. [222] with permission from Elsevier. (e) Succinimide formation and C‐termina lysine. Figure adapted from Bailey et al. [220] with permission from Taylor & Francis. (f) Phosphorylation. Figure adapted from van Schaick et al. [150] with permission from Elsevier. (g) Cyclization of N‐terminal Q. Figure adapted from Duivelshof et al. [237] with permission from Elsevier

**FIGURE 8:**
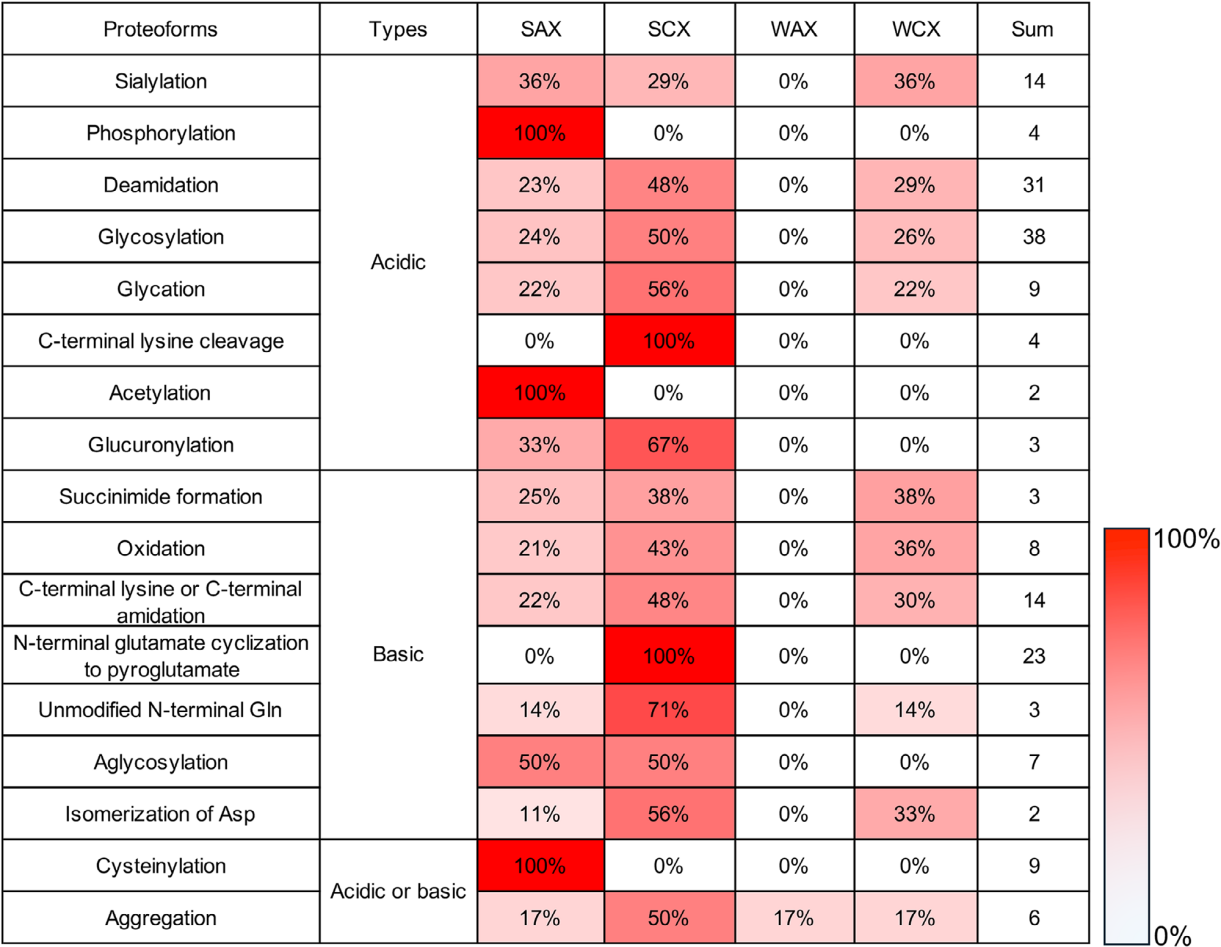
Summary and comparison of different IEC modes used to analyze charge variants covered in this review. The figure is made using the data from Tables 2, 3, 4, calculating the proportions of different PTMs analyzed by the four IEC modes of SAX, SCX, WAX, and WCX, respectively.

Figure 7 reports examples of separation and IEC analysis of various proteoforms. Figure 8, we summarize the different PTMs identified using IEC–MS methods (classified as acidic and basic according to Table 1), together with the type of IEC method applied (data from Tables 2, 3, 4). The more frequently used approach is SCX, reported in 26 of 53 studies. This prevalence is largely due to the focus on mAb‐based therapeutics, which typically have pI values above 7 and can therefore be readily retained under CEX conditions. In general, basic variants have been primarily studied using CEX methods, whereas acidic variants are typically analyzed using AEX methods. However, acid variants are also detected by CEX methods, particularly in cases such as sialylation, deamidation, C‐terminal lysine cleavage, and glucuronidation. Interestingly, strong ion exchange (SCX/SAX) methods are prevalent, whereas weak ion exchange methods are less commonly applied (e.g., only one WAX–MS is covered in this review). This presumably relates to the selectivity of the ion exchangers, the practical advantages of their charge independence of the applied pH, the physicochemical properties of analytes, and the range of analytical conditions that may facilitate method development, especially in pH‐mediated methods. Notably, while SAX is most commonly applied for detecting proteoforms such as phosphorylation, acetylation, and cysteinylation, SCX dominates the identification of C‐terminal lysine cleavage and N‐terminal glutamate cyclization to pE. WCX is generally applied to similar cases as SCX, but it is likely to be used for its different selectivity. Only one report was found describing WAX–MS for the analysis of human growth hormone aggregates.

**TABLE 1:** Overview of charge variants induced by different modifications.

Charge variants	Proteoforms/PTMs	Changes	References
Acidic	Sialylation (glycosylation)	Addition of sialic acid (COOH)	[161, 162, 163]
	Phosphorylation	Addition of phosphate groups (HPO_3_)	[164, 165]
	Deamidation (asparagine and glutamine)	COOH formation	[161, 166]
	Glycation	Loss of NH_2_	[167, 168, 169]
	C‐terminal (lysine) cleavage	Loss of NH_2_	[161, 170, 171]
	Acetylation	Loss of NH_2_	[172, 173, 174]
	Glucuronylation	COOH formation	[152]
Basic	Succinimide formation	Loss of COOH	[175, 176]
	Oxidation (methionine, cysteine, lysine, and histidine)	Conformational change	[40, 170]
	C‐terminal lysine or C‐terminal amidation	NH_2_ formation or loss of COOH	[161, 177, 178]
	N‐terminal glutamate cyclization to pyroglutamate Glu	Loss of COOH	[97, 163]
	Aglycosylation (loss of charged glycans)	Loss of COOH	[179, 180]
	Isomerization of aspartic acid (Asp)	Amide from α‐ to β‐carboxyl, sublet pI shift	[181, 182]
Acidic or basic	Cysteinylation	Formation of S─S bonds between cysteines	[183, 184, 185, 186, 187]
	Protein complexes formation and aggregation	Changes the exposed pI of the complexed or aggregated state due to alterations in the electrostatic environment and accessibility of charged residues	[188, 189, 190]

**TABLE 2:** Overview of method details (samples, separation conditions, mass spectrometry coupling approaches, and characterized variants) of recent publications (from 2010 to 2025) using pH/salt gradient‐based cation exchange–MS to characterize protein charge variants.

Category	Analytes	IEC mode	Mobile phases	Columns	Ionization approach	Charge variants observed	Year and reference
mAbs	mAb1, mAb2, and mAb3 (three different IgG2 mAb formulations)	WCX	A: 5 mM AmHy in 20% MeOH (pH 9.5) B: 5 mM AmHy in 20% MeOH (pH 10.5)	ProSwift WCX‐1S: 2.5 mm × 50 mm (monolithic column)	Organic modifiers in mobile phases	(1) Glycosylation; (2) aggregation (dimer)	2013, [143]
mAbs	Aged mAbs	SCX	A: 50 mM AmFo + FA (pH 3.9) B: 500 mM AmAc (pH 7.4)	MAbPac SCX‐10: 150 × 4 mm	Direct coupling	(1) Deamidation; (2) oxidation; (3) glycosylation	2017, [232]
mAbs	NISTmAb	SCX	A: 20 mM AmAc + 20 mM AA (pH 5.6) B: 140 mM AmAc + 10 mM AmBc (pH 7.4)	YMC‐BioPro SP‐F: 100 × 2.1 mm × 5 µm	Flow splitter	(1) N‐terminal Gln instead of pyroglutamic acid; (2) glucuronylation; (3) unprocessed C‐terminal Lys; (4) deamidation; (5) glycosylation	2018, [157]
mAbs	Trastuzumab, adalimumab, infliximab, bevacizumab, and cetuximab	SCX	A: 25 mM AmBc + 30 mM AA (pH 5.3) B: 10 mM AmHy + 2 mM AA (pH 10.18)	MAbPac SCX‐10 RS: 50 × 2.1 mm × 5 µm	Direct coupling	(1) Deamidation; (2) glycosylation; (3) C‐terminal lys	2018, [94]
mAbs	Trastuzumab	WCX	A: 50 mM AmAc (pH 6.6) B: 50 mM AmAc + AmHy (pH 10.1)	ProPac WCX‐10: 250 × 2.0 mm × 5 µm	Direct coupling	(1) Deamidation; (2) isomerization; (3) succinimide; (4) unclipped C‐terminal lysine; (5) sialylation; (6) glycosylation	2018, [220]
mAbs	CHO cell line expressed IgG1 kappa mAb (A mAb)	WCX	A: 20 mM AmFo (pH 6.5) B: 20 mM AmFo (pH 8.6)	ProPac WCX‐10: 250 × 4 mm × 10 µm	Direct coupling	(1) Glycosylation; (2) unprocessed C‐terminal lys variant; (3) deamidation; (4) oxidation; (5) succinimidation; (6) aggregation (dimer)	2018, [233]
mAbs	NIST IgG1 mAb, therapeutic mAbs 1, 2, and 3 (all IgG1)	SCX	A: 20 mM AmAc + AA (pH 5.6) B: 200 mM AmAc (pH 7.0)	BioResolve SCX: 100 × 2.1 mm × 3 µm	Direct coupling	(1) Free of C‐terminal lys; (2) N‐terminally pyroglutaminated; (3) a partially aglycosylated intact form; (4) glycosylation; (5) glycation	2019, [234]
mAbs	Adalimumab, infliximab, bevacizumab, cetuximab, trastuzumab, NISTmab, and rituximab	SCX	A: 25 mM AmBc + 30 mM AA (pH 5.3) B: 10 mM AmHy (pH 10.9)	MAbPac SCX‐10 RS: 50 × 2.1 mm × 5 µm	Direct coupling	(1) Without and with C‐terminal lys; (2) glycosylation; (3) succinimide formation; (4) glycation; (5) deamidation	2019, [219]
mAbs	Infliximab, trastuzumab, and NISTmAb	SCX	A: 20 mM AmAc + AA (pH 5.5) B: 140 mM AmAc + 10 mM AmBc (pH 7.4)	MAbPac SCX‐10 RS: 50 × 2.1 mm × 5 µm	Direct coupling	(1) Deamidation; (2) unprocessed C‐terminal lys	2020, [235]
mAbs	Cetuximab	SCX	A: 6.25 mM AmBc + 7.5 mM AA (pH 5.40) B: 5 mM AmHy (pH 9.85)	MAbPac SCX‐10 RS: 50 × 2.1 mm × 5 µm	Direct coupling	(1) Glycosylation; (2) C‐terminal lys; (3) non‐truncated C‐terminal lysine; (4) sialylation	2020, [218]
mAbs	NISTmab	SCX	A: 20 mM AmAc (pH 5.6, adjusted by AA) B: 150 mM AmAc (pH 6.8)	BioPro IEX SF column: 4.6 mm × 100 mm × 5 µm	Flow splitter Multi‐nozzle ESI DEN gas	(1) Glycosylation; (2) deamidation; (3) unremoved C‐terminal lys; (4) unconverted N‐terminal Q	2020, [151]
mAbs	Trastuzumab	WCX	A: 50 mM AmAc + AA (pH 5.0) B: 50 mM AmAc + AmHy (pH 9.5)	ProPac WCX‐10: 250 × 2 mm × 5 µm	Make up flow of organic modifiers	(1) High‐mannose glycosylation; (2) remaining C‐terminal lys residue; (3) Asn deamidation; (4) Asp isomerization; (5) oxidation (methionine and tryptophan)	2020, [146]
mAbs	Adalimumab, belimumab, dalotuzumab, eculizumab, elotuzumab, infliximab, ipilimumab, palivizumab, rituximab, NISTmab, and trastuzumab	SCX	A1: 20 mM AmAc (pH 5.6, adjusted by AA) B1: 140 mM AmAc + 10 mM AmBc (pH 7.4) A2: 10 mM AmAc + 10 mM AA B2: 50 mM AmAc + 25 mM AmCa A3: 50 mM AmAc + 2% ACN (pH 5.0) B3: 160 mM AmAc + 2% ACN (pH 8.5)	BioResolve SCX mAb: 50 × 2.1 mm × 3 µm MabPac SCX‐10 RS: 2.1 × 50 mm × 5 µm BioResolve SCX mAb: 4.6 × 50 mm × 3 µm	Organic modifiers in mobile phases	(1) Glycosylation; (2) pyroglutamate from N‐terminal Glu/Gln; (3) aglycosylation; (4) aspartate isomerization; (5) truncation of C‐terminal lys; (6) deamidation; (7) no N‐terminal modifications; (8) sialylation	2021, [97]
mAbs	Rituximab‐based biopharmaceuticals	SCX	A: 25 mM AmBc + 30 mM AA (pH 5.3) B: 10 mM AmHy (pH 10.8)	MAbPac SCX‐10 RS: 2.1 × 50 mm × 5 µm	Direct coupling	(1) C‐terminal lysine; (2) clipping of C‐terminal lys; (3) glycosylation; (4) glycation; (5) sialylated glycoforms	2021, [236]
mAbs	Emicizumab (IgG4, BsAb)	SCX	A: 50 mM AmAc in 2% ACN (pH 5.0) B: 160 mM AmAc in 2% ACN (pH 8.5)	BioResolve SCX mAb: 2.1 mm × 50 mm × 3 µm	Direct coupling	(1) Cyclization of the N‐terminal Gln; (2) glycosylation	2022, [237]
mAbs	Adalimumab, infliximab, pembrolizumab, mAb‐domain‐fusion, and mAb‐cytokine‐fusion	SCX	A: 50 mM AmAc (pH 5) B: 160 mM AmAc (pH 8.5) (mixed with different organic modifiers, 1% DMSO, 30% IPA, and 50 mM 1,1,1,3,3,3‐hexafluoro‐2‐propanol)	mAbPac SCX‐10 RS: 50 × 4.6 mm × 5 µm	Organic modifiers in mobile phases	None	2023, [145]
mAbs	Infliximab, pembrolizumab, adalimumab, bevacizumab, daratumumab, atezolizumab, denosumab, guselkumab, and rituximab	SCX	A: 25 mM AmBc (pH 5.3, adjusted by AA) B: 10 mM AmHy (pH 10.9)	Not mentioned	Direct coupling	(1) Glycation; (2) unprocessed C‐terminal lys	2023, [85]
mAbs	Eculizumab, trastuzumab, and pembrolizumab	SCX	A: 50 mM AmAc (pH 5.0) B: 160 mM AmAc (pH 8.6)	BioResolve SCX column: 2.1 × 100 mm × 3 µm	Direct coupling	(1) Deamidation; (2) isomerization of Asp	2023, [84]
mAbs	Bevacizumab and NISTmAb	SCX	A: 50 mM AmAc with 20% ACN (pH 5.5) B: 150 mM AmAc with 20% ACN (pH 8.5)	BioResolve SCX mAb column: 2.1 × 100 mm × 3 µm	Direct coupling	(1) Deamidation; (2) glycosylation; (3) un‐cleaved C terminal lys; (4) oxidation or N‐terminal glutamine; (5) aggregation	2023, [144]
mAbs	Pembrolizumab, cetuximab, and trastuzumab	SCX	A: 50 mM AmAc (pH 5.0, adjusted by AA) B: 250 mM AmAc (pH 8.5, adjusted by AmHy)	BioPro IEX SF based homemade capillary column: 150 mm × 75 or 100 µm × 5 µm	Nano‐flow IEC–MS	None	2024, [20]
Bispecific mAbs	Bispecific and mis‐paired antibody species	WCX	A: 50 mM AmAc (pH 7) B: 50 mM AmAc + 0.8% AmHy (pH 10)	ProPac WCX‐10 column: 4 mm × 250 mm × 10 µm	Direct coupling	None	2019, [227]
Bispecific mAbs	Therapeutic T‐cell bispecific humanized monoclonal antibody	WCX	A: 50 mM AmAc (pH 6.0) B: 50 mM AmAc (pH 10.7)	ProPac WCX‐10 column: 4 × 250 mm × 10 µm	Flow splitter	(1) Glycosylation; (2) deamidation; (3) sialic acid; (4) C‐terminal P‐amidation	2021, [154]
Bispecific mAbs	Bispecific antibody, and Fc‐infusion protein	WCX	A: 50 mM AmAc + AA (pH 5.0) B: 50 mM AmAc + AmHy (pH 9.5)	ProPac WCX‐10: 250 × 2 mm × 5 µm	Make up flow of organic modifiers	(1) High‐mannose glycosylation; (2) remaining C‐terminal lys residue; (3) Asn deamidation; (4) Asp isomerization; (5) oxidation (methionine and tryptophan)	2020, [146]
Bispecific mAbs	Bispecific antibody‐1, bispecific antibody‐2, monospecific antibody‐1, and monospecific antibody‐2	SCX	A: 20 mM AmAc (pH 5.6, adjusted by AA) B: 150 mM AmAc (pH 6.8)	BioPro IEX SF column: 4.6 mm × 100 mm × 5 µm	Direct coupling	(1) Glycation; (2) glucuronylation; (3) glycosylation; (4) N‐terminal Gln (non‐cyclized); (5) deamidation	2022, [238]
ADC	Antibody‐drug conjugate (trastuzumab‐AJICAP‐MMAE)	SCX	A: 50 mM AmAc (pH 5.0) B: 160 mM AmAc (pH 8.5)	BioResolve SCX mAb: 2.1 × 50 mm or 100 mm × 3 µm	Direct coupling	(1) Glycosylation	2020, [224]
ADC	Sigma MAb ADC Mimic	SCX	A: 20 mM AmAc (pH 5.6, adjusted by AA) B: 150 mM AmAc (pH 6.8)	BioPro IEX SF column: 4.6 mm × 100 mm × 5 µm	Flow splitter Multi‐nozzle ESI DEN gas	(1) Glycosylation; (2) deamidation; (3) unremoved C‐terminal lys; (4) unconverted N‐terminal Q	2020, [151]
Biopharmaceutical	N‐succinimidyl‐Sacetylthioacetate (SATA)‐conjugated lysozyme, reduced lysozyme, and stressed sample of interferon‐β	WCX	A: 100 mM AmAc (pH 7.0) B: 1 M AmAc (pH 7.0)	PolyCAT A column: 2.1 mm × 250 mm × 5 µm × 1000 Å (porous)	Direct coupling	(1) Disulfide bonds; (2) glycosylation; (3) deamidation	2015, [142]
Biopharmaceutical	PEGylated protein therapeutic: recombinant interferon‐β‐1a conjugated with a 20 kDa PEG chain	WCX	A: 100 mM AmAc B: 1 M AmAc	ProPac WCX‐10 column: 2 mm × 250 mm	Direct coupling	(1) Glycosylation; (2) PEGylation; (3) deamidation	2017, [230]
Biopharmaceutical	Bispecific antigen binding protein (BSaBP)	SCX	A: 50 mM AmAc (pH 5.2) B: 150 mM AmAc (pH 10.2)	YMC bio pro‐SPF‐IEX: 100 × 4.6 mm × 5 µm	Direct coupling	(1) Deamidation; (2) glycosylation; (3) aggregation (dimer); (5) isomerization; (6) succinimide; (7) oxidation	2024, [239]
Biopharmaceutical	RNase A, CA, BSA, and *Escherichia coli* cell lysates	SCX	A: 50 mM AmAc (pH 5.0, adjusted by AA) B: 250 mM AmAc (pH 8.5, adjusted by AmHy)	BioPro IEX SF based homemade capillary column: 150 mm × 75 or 100 µm × 5 µm	Nano‐flow IEC–MS	None	2024, [20]
Biopharmaceutical	SARS‐CoV‐2 recombinant vaccine	SCX	A: 25 mM AmBc (pH 5.3, adjusted by AA) B: 10 mM AmHy (pH 10.9)	MAbPac SCX‐10 RS column: 2.1 × 50 mm × 5 µm	Direct coupling	(1) Glycosylation	2024, [240]

*Note*: Nearly all of the columns are nonporous columns except for those with special notes.

Abbreviations: AA, acetic acid; ACN, acetonitrile; AmAc, ammonium acetate; AmBc, ammonium bicarbonate; AmCa, ammonium carbonate; AmFo, ammonium formate; AmHy, ammonium hydroxide; DMSO, dimethyl sulfoxide; FA, formic acid; IPA, isopropanol; MeOH, methanol.

**TABLE 3:** Overview of method details (samples, separation conditions, mass spectrometry coupling approaches, and characterized variants) of recent publications (from 2010 to 2025) using pH/salt gradient‐based anion exchange–MS to characterize protein charge variants.

Category	Analytes	IEC modes	Mobile phases	Columns	Ionization approach	Charge variants observed	Year and reference
mAbs	Seven in‐house mAbs (IgG1 and IgG4 subclasses) and NISTmA: mAb‐1 to mAb‐6 are IgG4‐based mAbs, mAb‐7 and NISTmAb are IgG1‐based mAbs	SAX	A1: 10 mM AmAc (pH 9.0, adjusted by AmHy) B1: 50 mM AmAc (pH 4.0, adjusted by AA) A2: 10 mM AmAc (pH 6.7) B2: 300 mM AmAc (pH 6.8)	YMC‐BioPro QA‐F SAX column: 4.6 mm × 100 mm × 5 µm	Flow splitter Multi‐nozzle ESI DEN gas	(1) Deamidation; (2) unprocessed C‐terminal lys; (3) glycation; (4) glycosylation; (5) succinimide; (6) noncyclized N‐terminal glutamine; (7) glucuronylation; (8) sialylation	2022, [152]
mAbs	IgG4	SAX	A1: 50 mM AmFo (pH 5.5) B1: 50 mM FA (pH 2.5) A2: 10 mM AmAc + 10 mM AmFo (pH 6.8) B2: 10 mM AA + 10 mM FA (pH 3.0)	ProPac SAX‐10 column: 2.0 × 250 mm × 10 µm	Flow splitter DEN gas	(1) Glycosylation; (2) phosphorylation	2023, [150]
Bispecific mAbs	Bispecific antibody‐1, bispecific antibody‐2, monospecific antibody‐1, and monospecific antibody‐2	SAX	A: 10 mM AmAc (pH 6.8) B: 300 mM AmAc (pH 6.8)	BioPro QA‐F SAX column: 4.6 mm × 100 mm × 5 µm	Direct coupling	(1) Deamidation; (2) C‐terminal lysine; (3) glycosylation	2022, [238]
Biopharmaceutical	Beta2‐microglobulin	SAX	35 mM AmFo (pH 7.4), and mixed with 0%, 10%, 20%, 30%, 50% ACN (isocratic elution)	ProPac SAX‐10: 2 mm × 250 mm	Direct coupling	(1) Aggregation	2013, [141]
Biopharmaceutical	Erythropoietin (EPO)	SAX	A: 30mM AmFo (pH 5.5) B: 30mM FA (pH 2.5)	Proteomix AEX‐NP5 column: 250 × 2.1 mm × 5 mm	Flow splitter	(1) Glycosylation; (2) sialylated forms; (3) deamidation; (4) acetylation; (5) phosphorylation	2021, [241]
Biopharmaceutical	Myozyme	SAX	A: 10 mM AmAc + 10 mM AmFo (pH 6.8) B: 10 mM AA + 10 mM FA (pH 2.9)	ProPac SAX‐10 guard column: 2 mm × 50 mm × 10 µm	Direct coupling	(1) Sialylation; (2) glycosylation	2023, [242]
AAV capsids	Research grade Sf9 derived standards of AAV 5, 6, and 8 full (CMV‐GFP) and empty material	SAX	A: 20 mM AmBc + 20 mM AmHy (pH 10.3) B: 15 mM FA + 30 mM AA (pH 2.6)	ProPac 3R column: 2.1 × 50 mm × 3 µm	Direct coupling	None	2023, [243]
Clinical sample	Human serum albumin	SAX	A: 50 mM AmAc (pH 7.4) B: 500 mM AmAc (pH 7.4)	Proteomix SAX‐NP5 column: 2.1 mm × 150 mm	Direct coupling	(1) Truncated C‐terminal leucine; (2) truncated N‐terminal residues; (3) glycation; (4) disulfide bridge; (5) deamidation; (6) oxidation; (7) sulfinic form of Cys residue; (8) cysteinylation	2018, [244]
Clinical Sample	Alpha‐1‐acid glycoprotein (AGP)	SAX	A1: 50 mM AmFo (pH 5.5, adjusted by FA) B1: 200 mM FA (pH 2.5) A2: 10 mM AmAc + 10 mM AmFo (pH 6.5) B2: 10 mM AA + 10 mM FA (pH 3.0)	ProPac SAX‐10 column: 2.0 mm × 250 mm × 10 µm	Flow splitter DEN gas	(1) Glycosylation; (2) sialylation; (3) pyroglutamate formation at the N‐terminal glutamine; (4) cysteinylation; (5) fucosylation	2024, [153]
Food Chemistry	Ovalbumin, goat anti‐human IgE‐HRP conjugate, and goat anti‐rabbit IgG‐HRP conjugate	SAX	A: 50 mM AmAc (pH 6.9) B: 500 mM AmAc (pH 6.9)	ProPac SAX‐10 column: 2.0 mm × 250 mm × 10 µm	Flow splitter	(1) Glycosylation; (2) acetylation; (3) phosphorylation; (4) deamidation; (5) aglycosylation; (6) oxidation	2021, [245]
Reference proteins	Ovalbumin	SAX	A: 10 mM AmAc + 10 mM AmFo (pH 6.8) B: 10 mM FA + 10 mM AA (pH 2.9)	ProPac SAX‐10 BioLC: 2 × 50 mm	Direct coupling	(1) Acetylation (N‐terminal); (2) deamidation; (3) phosphorylation; (4) succinimide formation; (5) sialylation; (6) glycosylation	2019, [202]
Reference proteins	Ovalbumin, RNase B, lipase, fetuin, and EndoPro	SAX	A1: 50 mM AmFo (pH 5.5) B1: 50 mM FA (pH 2.5) A2: 10 mM AmAc and 10 mM AmFo (pH 6.8) B2: 10 mM AA and 10 mM FA (pH 3.0)	ProPac SAX‐10 column: 2.0 × 250 mm × 10 µm	Flow splitter DEN gas	None	2023, [150]
Reference proteins and clinical samples	Reference proteins: Carbonic anhydrase, ovalbumin, enolase, cytochrome c, and lysozyme. Clinical samples: Human heart tissue lysate	Mixed‐bed IEC (WAX‐WCX)	A: 10 mM AmAc B: 800 mM AmAc	PolyCATWAX column: 0.3 × 100 mm × 3 µm × 1500 Å (porous)	Direct coupling	None	2024, [131]

*Note*: Nearly all of the columns are nonporous columns except for those with special notes.

Abbreviations: AA, acetic acid; ACN, acetonitrile; AmAc, ammonium acetate; AmBc, ammonium bicarbonate; AmCa, ammonium carbonate; AmFo, ammonium formate; AmHy, ammonium hydroxide; DMSO, dimethyl sulfoxide; FA, formic acid; IPA, isopropanol; MeOH, methanol.

**TABLE 4:** Overview of method details (samples, separation conditions, mass spectrometry coupling approaches, and characterized variants) of recent publications (from 2010 to 2025) using pH/salt gradient‐based 2DLC to characterize protein charge variants.

Category	Analytes	IEC modes	Mobile phases	Columns	2DLC configuration	Ionization approach	Charge variants observed	Year and reference
mAbs	IgG4 (MAb1) and IgG1 (MAb2 and MAb3) subtypes	WCX	A1: 20 mM Aces buffer (pH 6.1) B1: 50 mM NaCl in A1 A2: 10 mM sodium phosphate buffer (pH 7.5) B2: 100 mM NaCl in A2	ProPac WCX‐10 column: 4.0 × 250 mm	WCX × RPLC (multiple heart‐cut)	Direct coupling	(1) Glycosylation; (2) sialylation; (3) uncylized glutamine residue; (4) substitution of serine (Ser) to arginine (Arg)	2011, [163]
mAbs	Rituximab	WCX	A: 10 mM MES buffer (pH 5.5) B: 500 mM NaCl + A	Agilent Bio Mab NP5 column: 2.1 × 250 mm × 5 µm	WCX × RPLC (comprehensive)	Direct coupling	(1) Glycosylation; (2) C‐terminal lysine; (3) oxidation; (4) disulfide bonds; (5) sialylation	2015, [140]
mAbs	Trastuzumab, cetuximab; infliximab, infliximab‐B, trastuzumab‐B, and cetuximab‐B	WCX	A: 10 mM AmAc (pH 6.0) B: 250 mM AmAc (pH 6.0)	Agilent Bio Mab NP5 column: 2.1 × 250 mm × 5 µm	WCX × RPLC (comprehensive)	Direct coupling	(1) Missing the C‐terminal lysine; (2) glycosylation; (3) sialylation; (4) cyclization of N‐terminal glutamate to pyroglutamate (pE); (5) deamidation	2016, [246]
mAbs	Reslizumab, and bevacizumab	WCX	A: 20 mM AmAc (pH 5.0) B: 1 M AmAc (pH 5.5)	ProPac Elite WCX column: 2 × 50 mm	SEC × WCX (multiple heart‐cut)	Direct coupling	(1) Aggregation	2022, [247]
mAbs	IgG1 (kappa mAb, mAb‐A)	SCX	IonHance CX‐MS A and B buffers (diluted 1:10)	SCX BioResolve: 2.1 × 50 mm × 3 µm	SEC × SCX (multiple heart‐cut)	Direct coupling	(1) Glycosylation; (2) oxidation	2022, [222]
mAbs	IgG1 mAb	WCX	A: 25 mM AmAc (pH 6.0) B: 100 mM AmAc (pH 9.0)	Agilent Bio WCX column NP5: 4.6 × 250 mm × 5 µm)	HIC × WCX (multiple heart‐cut)	Direct coupling	(1) Oxidation; (2) glycation; (3) deamidation; (4) C‐terminal lys; (5) pyroglutamic acid; (6) thiol variant; (7) succinimidation	2022, [221]
mAbs	Biosimilar of trastuzumab, rituximab, and adalimumab	SCX	A: 15 mM AmAc (pH 6.8) B: 100 mM AmAc (pH 9.0)	Agilent Bio SCX NP5 column: 4.6 × 250 mm × 5 µm	SCX × SAX (multiple heart‐cut)	Direct coupling	(1) Deamidation; (2) isomerization of Asp; (3) glycosylation; (4) pyroglutamic acid; (5) C‐terminal lys	2023, [223]
mAbs	mAb A (IgG1 subclass)	WCX	A: 25 mM AmAc (pH 6.0) B: 100 mM AmAc (pH 9.0)	Agilent Bio Mab column NP5: 2.1 × 250 mm × 5 µm	SEC × WCX (multiple heart‐cut)	Direct coupling	(1) Glycosylation; (2) deamidation; (3) glycation; (4) oxidation (methionine)	2023, [248]
mAbs	Trastuzumab and biosimilars (Biceltis, Canmab, Trasturel, and Vivitra)	SCX	A: 15 mM AmAc (pH 6.8) B: 100 mM AmAc (pH 9.0)	Agilent Bio SCX NP3 SS column: 4.6 × 50 mm × 3 µm	ProA × SCX (multiple heart‐cut)	Direct coupling	(1) Deamidation; (2) isomerization; (3) succinimide	2024, [249]
mAbs	Co‐formulated antibodies: mAb A and mAb B	WCX	A: 20 mM phosphate buffer (pH 6.6) B: 500 mM NaCl + A (pH 6.6)	ProPac WCX‐10 BioLC analytical column: 4 × 250 mm × 10 µm	HIC × WCX × RPLC (multiple heart‐cut)	Direct coupling	(1) Carboxymethylation; (2) deamidation; (3) C‐terminal lysine; (4) VHS‐containing variant; (5) glycosylation; (6) isomerization; (7) sialylation	2024, [139]
mAbs	Unstressed and stressed humanized IgG1	SCX	A: 20 mM MES (pH 5.8) B: 300 mM NaCl + A	MabPac SCX‐10 column: 2 × 250 mm	SCX × FcRn (multiple heart‐cut)	Direct coupling	(1) Glycosylation; (2) oxidation; (3) afucosylation; (4) deamidation	2024, [250]
ADC	ADC‐S01, and thermally stressed ADC‐S01 for either 2 or 4 weeks	SCX	A: 20 mM MES (pH 6.5) B: 300 mM NaCl + A	BioResolve SCX column: 2.1 ×100 mm × 3 µm	SCX × RPLC (comprehensive)	Direct coupling	(1) Oxidation; (2) drug modifications	2022, [138]
mAbs	Biosimilar of trastuzumab, rituximab, and adalimumab	SAX	A: 15 mM AmAc (pH 9.5) B: 100 mM AmAc (pH 4.5)	Agilent Bio SAX NP5 column: 4.6 × 250 mm × 5 µm	SCX × SAX (multiple heart‐cut)	Direct coupling	(1) C‐terminal amidation; (2) isomerization of Asp; (3) deamidation; (4) glycosylation; (5) oxidation	2023, [223]
AAV capsids	Empty and full capsids of three AAV serotypes (AAV8, AAV5, and AAV1)	SAX	A: 20 mM bis‐tris propane (pH 9.5, adjusted by HCl) B: 20 mM bis‐tris propane + 1 M of either TMAC or TEAC (pH 9.5, adjusted by HCl)	ProPac SAX‐10 column: 2.0 × 250 mm × 10 µm	SAX × RPLC (multiple heart‐cut)	Direct coupling	None	2022, [137]
Biopharmaceutical	Protein human growth hormone	WAX	A: 20 mM AmAc (pH 5.0) B: 1 M AmAc (pH 5.5)	ProPac WAX‐10 column: 2 × 50 mm	SEC × WAX (multiple heart‐cut)	Direct coupling	(1) Aggregation	2022, [247]
Reference proteins and clinical samples	Reference proteins: Enolase, alcohol dehydrogenase, ovalbumin, and trypsinogen. Clinical samples: Endogenous protein complexes from human heart tissue	Mixed‐bed IEC (WAX‐WCX)	A: 10 mM AmAc B: 500 mM AmAc	PolyCATWAX column: 2.1 × 100 mm × 3 µm × 1500 Å (porous)	SEC × mixed‐bed IEC (multiple heart‐cut)	Direct coupling Flow splitter	None	2025, [132]

*Note*: Nearly all of the columns are nonporous columns except for those with special notes.

Abbreviations: Aces, *N*‐(2‐acetamido)‐2‐aminoethanesulfonic acid; AmAc, ammonium acetate; HCl, hydrochloric acid; MES, 2‐(*N*‐morpholino) ethanesulfonic acid; NaCl, sodium chloride; TEAC, tetraethylammonium chloride; TMAC, tetramethylammonium chloride; Tris, Tris‐(hydroxymethyl)‐aminomethane.

### 4.1 Biopharmaceuticals Analysis

Biopharmaceuticals have emerged as critical therapeutic agents, enabling the treatment of complex diseases such as cancer and autoimmune disorders [211, 212, 213]. Among these, recombinant mAbs are one of the most prominent products thanks to their high specificity, efficacy, and extended half‐life [214, 215]. mAbs are heterogeneous and characterized by the presence of various proteoforms (e.g., PTMs such as glycosylation) [216, 217]. As proteoforms can significantly affect a product's activity, their presence and abundance (e.g., glycoform profile) can be part of the critical quality attributes (CQAs) of a product. CQAs must be assessed to ensure consistent quality monitoring during manufacturing.

During product development and quality control, IEC serves as a critical analytical tool for assessing PTMs at the intact protein level and for monitoring these as CQAs, since many PTMs alter the charge properties of proteins. It allows us to obtain mass information directly related to the observed charge variants, thereby eliminating the tedious fractionation required for IEC separation before MS characterization.

#### 4.1.1 mAb‐Based Therapeutics

One of the first methods to be described was the application of IEC–MS to characterize the charge variants of mAb after long‐term storage, analyzing the charge variants using middle‐up analysis under native MS conditions (Table 2). This work was followed up shortly after by Füssl et al., who demonstrated the use of pH gradient elution with volatile and low ionic strength for direct coupling to MS, allowing the analysis of a variety of mAb products [94]. This provided, for the first time, online intact protein IEC–MS evidence of lysine truncation and possibly deamidation. Later on, the same authors applied SCX–MS to identify more than 16 different adalimumab charge variants caused by 7 different PTMs and explored the high structural heterogeneity (e.g., Fc and Fab glycosylation, sequence, and sialylation) variants of cetuximab isoforms [218, 219].

It is challenging to identify the deamidation at the intact protein level by IEC–MS due to the minor MW alteration (e.g., 1 Da) with respect to the MW of the protein (e.g., about 150 kDa for a mAb). Murisier et al. suggested that deamidation may not be unambiguously identified by SCX–MS alone [97]. Furthermore, achieving high‐precision mass measurements for these subtle mass differences requires an adequate abundance of the isoform, a point emphasized by Bailey et al. [220]. Therefore, to confirm the presence of such modifications and map their locations, a necessary follow‐up step is to fractionate the IEC peak and perform a bottom‐up peptide analysis.

Recent progress in IEC–MS was shown by the work of Yan et al., where ultrasensitive SCX–MS allowed the characterization of NISTmAb at both antibody and subdomain levels. Especially, the Fab‐glycosylated variants present at a very low level (< 0.1%) can be directly detected without any enrichment [157]. Moreover, to investigate the conformational landscape of mAbs that may be affected by PTMs, Schaick et al. developed the IEC–CIU (collision‐induced unfolding) method to monitor the unfolding pattern of mAbs at the intact level. They successfully characterized the conformational properties of mAb glyco‐variants [84].

For mAb‐based therapeutics, generally, the SCX‐based method is typically used. However, Liu et al. demonstrated that AEX–MS (SAX) can also be used to separate charge variants of IgG4, which shows excellent resolving power for multiple attributes in the IgG4 Fc region and a complementary selectivity to SCX methods [152]. In addition, 2DLC–MS methods have been developed to characterize mAb‐based therapeutics including IEC as separation dimension prior MS (Table 4). Examples include the work of Sarin et al. who created a HIC–WCX method to identify the hydrophobic and charge variants for two mAbs [221], Lambiase et al. utilized SEC–SCX to monitor the size and charge variants of one mAb [222], and Kumar developed SCX‐SAX to separate acidic, main species, and basic variants simultaneously [223].

Other therapeutics that have also been studied with IEC–MS methods include: (i) antibody‐drug conjugate (ADC), (ii) co‐formulated antibodies (CFAs), (iii) bispecific antibodies (BsAbs), (iv) single domain antibodies (sdAbs), and (v) PEGylated proteins. (i) ADCs are composed of mAb linked with a small molecule drug through covalent chemical conjugation. Matzuda et al. successfully analyzed intact native ADC to determine the drug‐antibody ratio, charge variants status, and glycan profile in a single run with SCX–MS [224]. (ii) CFAs are pharmaceutical products that combine two or more antibodies in a single dosage form, aiming to improve therapeutic efficacy and target more antigens compared with single mAb [225, 226]. To separate each charge variant of the two antibodies simultaneously, Jin et al. developed a 3DLC–MS (HIC–IEC–RPLC) method to collect CFA (HIC), separate charge variants (IEC), and perform online desalting (RPLC), thereby achieving the simultaneous analysis of charge variant ratios and PTM information [139]. (iii) BsAbs combine the characteristics and specificities of two different antibodies to target two distinct antigens in a single moiety. WCX–MS is a good strategy to investigate the intrinsic charge, size, isobars, and shape differences of these molecules [154, 227]. (iv) sdAbs are valuable probes for molecular imaging due to their long blood retention. They are the smallest antigen‐binding domain (around 15 kDa) derived from heavy chain‐only antibodies, functionalized with different degrees of conjugated chelators on lysine sites to make probes [228]. The AEX–MS helps separate and identify these complex samples to determine the impact of conjugation degree [136]. Finally, (v) protein PEGylation was proven to effectively enhance the pharmacokinetic and pharmacodynamics of protein therapeutics due to their resistance to proteolytic degradation, lower renal clearance, and less immunogenic response [229]. IEC–MS offers a reliable method for resolving positional isomers, assessing the degree of PEGylation, and identifying modified patterns [230, 231].

#### 4.1.2 Viruses and Vaccines

AAVs are non‐enveloped, single‐stranded DNA viruses that are able to transduce a diversity of species in vivo with low immunotoxicity risks [251, 252]. They have been applied to gene therapy applications such as a monotherapy or other biomolecules [253, 254]. AAV capsid consists of three types of viral proteins (VPs) with an MW of megadalton, and it has different VP compositions and PTMs based on the serotypes [255, 256]. AAV products are usually composed of a mixture of full, partially filled, and empty capsids for gene therapy, which include different amounts of negatively charged DNA [257]. The separation of different extents of encapsulated AAVs and the identification of their PTMs are vital to evaluating the tropism, infectivity, and transduction efficiency. Wu et al. utilized a SAX–RPLC–MS platform to separate empty and full capsids in AAV samples and characterize the specific PTMs of VPs [137]. Noroviruses are non‐enveloped, positive‐strand RNA viruses that pose a big threat to public health. They have a common capsid structure with 180 copies of the major capsid protein VP1, of which the C‐terminus forms a protruding (P)‐domain to mediate receptor attachment [258]. Creutznacher et al. found an irreversible deamidation in Asn373 located in P‐domain proteins, resulting in a negative charge in each monomer [258]. They used IEC and offline native MS to investigate the kinetics of monomer exchange based on the charge differences between modified Asn373 and point‐mutated Asn373Gln. Finally, Wu et al. employed SCX–MS and imaged capillary isoelectric focusing (icIEF) to identify the charge variants of SARS‐CoV‐2 recombinant vaccine at the intact level and integrated other techniques to conduct an in‐depth analysis of their heterogeneity [240].

### 4.2 Endogenous Proteoforms Analysis

PTMs, such as glycosylation, often affect the stability, activity, and function of proteins. Investigating these native PTM forms can provide a deeper understanding of the relationship between structure and function, yet to date, only limited studies have applied IEC–MS to analyze endogenous proteins.

Füssl et al. used pH gradient‐based SAX‐MS method to analyze serum albumin, human transferrin, and natural human alpha‐1‐acid glycoprotein (AGP) [202]. Following up on this initial study, van Shaick et al. applied an AEX–MS method to study pregnancy‐associated changes in AGP proteoforms [153]. This approach revealed over 400 proteoforms, including highly sialylated higher antennary structures averaging 16 sialic acids and 0–1 fucose per protein; notably, AGP1 variants exhibited slightly higher fucosylation than AGP2. Their proteoform assignment was supported by integrating exoglycosidase‐treated samples and glycopeptide analysis after tryptic digestion.

Cramer et al. described the analysis of plasma proteoforms combining offline native IEC (SCX) separations with offline native MS analysis [259]. To achieve this, they first depleted the serum from the most abundant proteins and collected fractions every 0.5 min over about 20 min of the SCX gradient. With this workflow, they could observe over 20 serum (glyco)proteins with MWs of 30–190 kDa for each donor serum samples. In addition, they analyzed serum samples from both healthy and diseased individuals to monitor the proteoform profiles. Subsequently, they found a rapid increase in fucosylation and glycan occupancy in patients compared to healthy individuals.

High‐density lipoproteins (HDLs) were also analyzed by IEC–MS analysis. The level of these proteins is believed to have a strong inverse relationship with coronary heart disease [260]. HDL also has antioxidant and anti‐inflammatory activity [261]. It consists of 50% protein and 50% lipid by mass and is divided into three types according to density, namely, HDL2 and HDL3 [262]. Instead of using the traditional density‐gradient ultracentrifugation method, Hsieh et al. used AEX and MALDI–MS to characterize HDLs from six healthy donors with increasing charges and evaluate their composition and biological activities [262].

Beyond targeted proteoform study, separating proteoforms within a complex sample using a single method presents a significant challenge. To address this, Fischer et al. developed an online mixed‐bed ion exchange column to enable separation of acidic and basic endogenous proteins simultaneously from complex mixture under non‐denaturing conditions, preserving non‐covalent interactions for native top‐down proteomics analysis [131, 132]. In addition, Zhai et al. developed a highly sensitive nano‐flow SCX–nMS method for the direct analysis of *Escherichia coli* cell lysates [20]. This allowed them to identify over 100 species with a high dynamic range of intensities and molecular weights ranging from 10 to 150 kDa. Notably, proteoforms differing by as little as 54 Da, with molecular weights up to 141 kDa, were successfully separated. This setup demonstrates significant potential for analyzing complex native samples at the intact protein level and for other sample‐limited applications.

### 4.3 Food Protein Analysis

IEC–MS could play a crucial role in the food industry by identifying specific proteins present in food and their corresponding isoforms, thereby contributing to enhanced food quality and safety (e.g., studying potential links between allergies and proteoforms present). For instance, osteopontin (OPN), an acidic and highly phosphorylated glycoprotein involved in various physiological functions, is a potential candidate ingredient in infant formula due to its ability to mimic breast milk [263, 264] and promote infant gut health and brain development [134]. OPN is an acidic protein, therefore, AEX method is suited for its characterization. To date, however, only AEX–UV methods have been developed [134].

Ovalbumin is another example. This protein is extensively used in the food industry for its high nutritional value and excellent functional attributes. It is also a major human allergen, with its allergenicity linked to glycosylation (N292) and phosphorylation (S68 and S344). Yang et al. utilized SAX–MS to assess the structural heterogeneity of native ovalbumin. They found that proteoforms associated with acetylation, phosphorylation, oxidation, and succinimide modifications reduced its immunoglobulin G (IgG)/immunoglobulin E (IgE) binding capacities, while sialylation was beneficial to this binding capacity [245]. Furthermore, Füssl et al. developed a pH gradient‐based SAX–MS method to characterize ovalbumin proteoforms at the intact protein level under native conditions, identifying over 150 different proteoforms, including fragments and dimers [202].

## 5 Summary and Outlook

In this review, we highlight the pH gradient‐based IEC–MS techniques for characterizing a diversity of proteins and their variants from the intact level. Compared with salt gradient methods, the pH gradient and salt‐mediated pH gradient approaches can provide a simple way to achieve a direct coupling between IEC and MS using volatile additives (e.g., AmAc). IEC–MS is also considered a native chromatographic mode, creating opportunities to preserve the high‐order structure, integrity of molecules, and original compositions of proteins. This method offers excellent resolving power, unique charge‐based selectivity, compared with other native separation techniques. Many basic or acidic charge variants of proteins induced by diverse PTMs, such as glycosylation, deamidation, oxidation, and C‐terminal lysine, can be investigated by different IEC modes (coupled with MS). Moreover, they have been widely applied to identify a plethora of proteins and proteoforms, including mAbs, bispecific mAbs, ADCs, virus, vaccine, cell lysates, and the clinical samples.

New strategies have been recently introduced to address some of the challenges in IEC–MS. For instance, three approaches (novel volatile salts, correction of nonlinear pH profile, and salt‐assisted fast elution in the linear pH response zone) are developed to solve the rapid pH change‐induced co‐elution of analytes due to the limited choice of volatile salts. Organic modifiers, multi‐nozzle ESI, dopant‐enriched nitrogen gas, and nano‐flow IEC–MS are employed to enhance ionization and increase sensitivity. Although some techniques are still under development and improvement, they point to some promising directions for IEC–MS and are expected to establish a solid foundation for intact proteoform analysis, opening new opportunities in biopharmaceutical analysis, native top‐down proteomics, clinical studies, and other sample‐limited applications.

## Author Contributions

**Ziran Zhai**: conceptualization, investigation, writing – original draft, writing – review and editing. **Thomas Holmark**: writing – original draft, writing – review and editing. **Lars Jasperse**: writing – original draft. **Andrea F. G. Gargano**: conceptualization, investigation, project administration, funding acquisition, supervision, writing – review and editing.

## Conflicts of Interest

The authors declare no conflict of interest.

## Data Availability

Data sharing is not applicable to this article as no datasets were generated or analyzed during the current study.
